# Building the Future of Transportation: A Comprehensive Survey on AV Perception, Localization, and Mapping

**DOI:** 10.3390/s25072004

**Published:** 2025-03-23

**Authors:** Ashok Kumar Patil, Bhargav Punugupati, Himanshi Gupta, Niranjan S. Mayur, Srivatsa Ramesh, Prasad B. Honnavalli

**Affiliations:** Department of Computer Science Engineering, PES University, Bangalore 560085, India; bhargav.punugupati@gmail.com (B.P.); himanshig1403@gmail.com (H.G.); niranjanmayur10@gmail.com (N.S.M.); vatsa2402@gmail.com (S.R.); prasadhb@pes.edu (P.B.H.)

**Keywords:** autonomous vehicles, object detection, tracking algorithms, localization, mapping, SLAM, perception module, HD maps, sensor fusion, LiDAR

## Abstract

Autonomous vehicles (AVs) depend on perception, localization, and mapping to interpret their surroundings and navigate safely. This paper reviews existing methodologies and best practices in these domains, focusing on object detection, object tracking, localization techniques, and environmental mapping strategies. In the perception module, we analyze state-of-the-art object detection frameworks, such as You Only Look Once version 8 (YOLOv8), and object tracking algorithms like ByteTrack and BoT-SORT (Boosted SORT). We assess their real-time performance, robustness to occlusions, and suitability for complex urban environments. We examine different approaches for localization, including Light Detection and Ranging (LiDAR)-based localization, camera-based localization, and sensor fusion techniques. These methods enhance positional accuracy, particularly in scenarios where Global Positioning System (GPS) signals are unreliable or unavailable. The mapping section explores Simultaneous Localization and Mapping (SLAM) techniques and high-definition (HD) maps, discussing their role in creating detailed, real-time environmental representations that enable autonomous navigation. Additionally, we present insights from our testing, evaluating the effectiveness of different perception, localization, and mapping methods in real-world conditions. By summarizing key advancements, challenges, and practical considerations, this paper provides a reference for researchers and developers working on autonomous vehicle perception, localization, and mapping.

## 1. Introduction

Autonomous vehicles (AVs) are poised to transform transportation by improving safety, efficiency, and accessibility. A crucial component of AV technology is the perception system, which enables vehicles to interpret their environment and make informed navigation decisions. However, real-world driving conditions pose numerous challenges, including occlusion, sensor limitations, and real-time processing constraints. Although previous surveys have reviewed perception, localization, and mapping methodologies, our study differentiates itself by not only analyzing existing research but also testing and evaluating various approaches for object detection, tracking, localization, and mapping in realistic scenarios.

This paper aims to comprehensively assess AVs’ perception, localization, and mapping techniques. We begin by analyzing the architecture and workflow of these modules, explaining how they interact to facilitate safe navigation. A detailed evaluation of existing datasets follows, focusing on their diversity, sensor configurations, and suitability for real-world applications. We then assess state-of-the-art object detection models such as You Only Look Once version 8 (YOLOv8) [1], Single Shot MultiBox Detector (SSD) [2], Faster Region-based Convolutional Neural Network (Faster R-CNN) [3], and tracking algorithms like DeepSORT [4], ByteTrack [5], Kalman filtering [6], and BoT-SORT (Boosted SORT) [7], examining their robustness under conditions such as occlusion, varying lighting, and dynamic traffic environments. Furthermore, we investigate LiDAR-based, vision-based, and sensor fusion-based localization strategies, discussing their accuracy and effectiveness in scenarios where Global Positioning System (GPS) signals are unreliable or unavailable. The mapping component of our study delves into Simultaneous Localization and Mapping (SLAM) [8] and high-definition (HD) maps [9], evaluating their role in creating real-time, dynamic environmental representations for AV navigation.

Unlike many studies that rely solely on literature reviews, this paper integrates empirical insights from our experimental testing. We implemented object detection and tracking pipelines, evaluated different localization techniques, and tested mapping methods using high-resolution cameras, light detection and range (LiDAR) sensors, inertial measurement units (IMUs), and other sensor technologies. Our experiments assess the performance of these algorithms under real-world conditions, including adverse weather, occlusion, and urban traffic complexities. Synthesizing theoretical advances with practical testing offers a realistic perspective on what works best for AV perception and navigation.

This paper is structured into eight sections, each addressing a fundamental component of AV perception, localization, and mapping. Section 2 explores object detection and tracking, detailing the evolution of convolutional neural networks (CNNs) in AV perception and comparing one-stage and two-stage detection models. We discuss how tracking algorithms such as DeepSORT [4], BoT-SORT [7], and ByteTrack [5] maintain object identity over time, particularly in challenging scenarios. Section 3 focuses on key object detection and tracking challenges, including occlusion, adverse weather conditions, and nighttime performance. Section 4 reviews commonly used datasets such as KITTI, nuScenes, and Waymo Open Dataset, evaluating their sensor configurations and annotation quality. Moving beyond perception, Section 5 examines localization techniques, comparing vision-based, LiDAR-based, and sensor fusion approaches and their effectiveness in achieving precise vehicle positioning. Section 6 explores different mapping technologies, discussing traditional digital maps, HD maps, and SLAM-based real-time mapping. As an alternative approach, Section 7 investigates the emerging concept of mapless navigation, which reduces dependency on prebuilt maps by leveraging SLAM, visual odometry, and deep learning techniques. Finally, Section 8 synthesizes our findings, discussing the remaining challenges in AV perception and mapping while outlining potential future research directions, such as improving real-time processing efficiency, optimizing sensor fusion strategies, and developing adaptive mapping techniques.

This paper integrates insights from the literature with experimental evaluations, serving as a practical reference for researchers, engineers, and industry professionals working on the perception, localization, and mapping of AV. Our goal is to bridge the gap between theoretical advancements and real-world implementations, ensuring that AV systems are robust in simulation environments and capable of functioning reliably in diverse and dynamic real-world scenarios.

## 2. Object Detection and Tracking in Autonomous Vehicles

### 2.1. Introduction to Image Processing in Autonomous Vehicles

**Figure 1:**
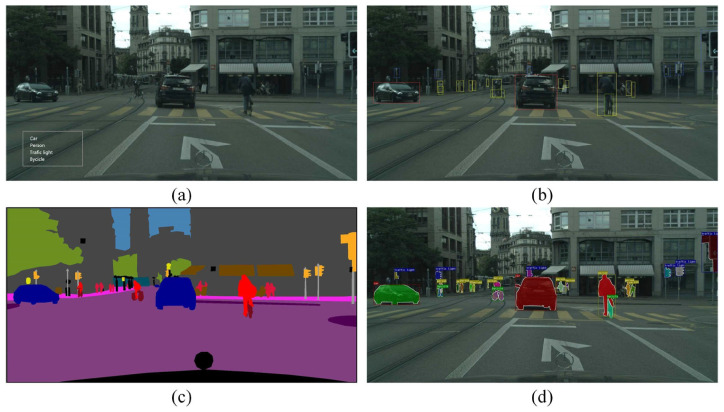
Relationship between different vehicle detection algorithms: (**a**) object classification, (**b**) object detection, (**c**) semantic segmentation, (**d**) instance segmentation. Source: [10].

Autonomous vehicles rely heavily on image processing to understand and interact with their environment. The journey begins with image classification, where various features within an image are identified and categorized, such as classifying an object as a “car” or “pedestrian”. However, image classification alone is insufficient for AVs, as they need to know where objects are located in the scene. This leads to object detection, which involves identifying and localizing objects within an image, often represented by bounding boxes around the objects. Moving further into the image processing chain, the segmentation of the image provides a more detailed understanding by classifying each pixel in the image, which is crucial for accurate scene understanding in complex environments like roads with multiple moving objects, as shown in Figure 1.

### 2.2. Overview of Convolutional Neural Networks (CNNs)

Convolutional Neural Networks (CNNs) [11] form the backbone of modern image processing and object detection in AVs. A CNN comprises multiple layers, each performing a specific function to process an input image and extract meaningful information. An array of input signals is transformed into an array of output signals in each convolutional layer. Each output is called a feature map. If the input is a picture, this feature map will be a 2D array of color channels; if the input is a video, this feature map will be a 3D array; and for audio input, it will be a 1D array.

- Input layer: Larger structures are introduced to a network by input layers, which are normally represented by an image consisting of a multi-dimensional array of pixels. When images are to be put in a model, they should conform to the region, channel dimensions, and the model or deep learning library.
- Convolution layer: while a kernel specifies the view size in the convolution operation, a filter, regarded as a tensor in this occasion, is the total number of kernels in a given layer channel-wise, for example, a filter with m×n×c dimensions, has c pieces of m×n kernels.
- Padding: Using this mechanism, the picture is resized in the spatial dimension, and the number of channels depends on both the input and the filter size. Parameters of the kernel define features that present edges in the output that are relevant to those characteristics.
  Padding adds a number of pixel grids to extend the spatial plane’s output dimensions. It also addresses the common problem of information loss. Thus, padding is a critical factor in convolutional neural network architecture and performance.
- Stride: stride relates to the translation distance in pixels when convolving the images.
- Activation functions: A feature of the network requiring more dense representation has its respective feature map built via the activation function, transforming the feature map into a nonlinear zone and allowing the model to learn complex tasks over data. The model can be made for more complicated structures by permuting the straight surfaces. For relief, the famous option for CNNs defeats the vanishing gradient problem.
- Pooling operation: The pooling layers in CNNs remove small rectangular structures from the output feature map, and each small block chooses a single maximum, minimum, or average number. With this adjustment, the feature map’s parameters and spatial size are smaller, making it easier to aim at microregulation. There are different sorts of pooling, such as maximum pooling or global average pooling. The second is when the whole image is pooled but nothing undergoes a sliding operation.
- Fully Connected Layers: after completing the convolutional and pooling processes, the last part of the CNN structure is the Fully Connected Layers (FCLs) used for high-level classification.

CNNs excel at handling complex tasks like object detection and tracking, enabling AVs to process real-time information with high accuracy.

### 2.3. Object Detection Networks

**Figure 2:**
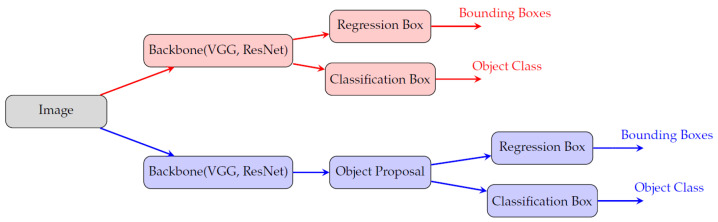
Difference between one-stage and two-stage object detection. (Red color is for single stage detection and blue color is for two stage detection.

Object detection involves recognizing objects and pinpointing their locations within an image. Several CNN-based architectures have been developed to achieve efficient and accurate object detection in real time. These architectures are typically categorized into two-stage and one-stage detectors, as given in Figure 2.

#### 2.3.1. Two-Stage CNN Detectors

Two-stage CNN detectors have been integral to the evolution of object detection, beginning with R-CNN (Region-based Convolutional Neural Network), introduced in 2014 by Girchick et al. [12] This model relies on region proposals, which are processed individually through a backbone network, such as AlexNet. Despite achieving a mean Average Precision (mAP) of 58.5% on the Pascal VOC dataset, R-CNN suffered from slow processing speeds (one frame per second) and high computational demands, making it impractical for real-time applications.

In 2015, SPP-Net (Spatial Pyramid Pooling Network), proposed by He et al. [13], introduced spatial pyramid pooling to reduce redundant processing of region proposals, improving computational efficiency. SPP-Net achieved an improved mAP of 60.9% on the Pascal VOC dataset, but the multi-stage training process remained a drawback.

Later in 2015, Girshick [14] introduced Fast R-CNN, which brought significant improvements by incorporating a Region of Interest (RoI) pooling layer, leading to faster training and testing. This model achieved up to a 70.0% mAP on the Pascal VOC dataset, offering advantages like end-to-end training and improved accuracy compared to earlier models.

By 2016, Faster R-CNN, developed by Ren et al. [3], further improved on its predecessors by integrating a Region Proposal Network (RPN) to generate region proposals directly from the image, bypassing external proposal algorithms. Faster R-CNN provided faster and more accurate detection due to the unified proposal generation and classification process.

In the same year, R-FCN (Region-based Fully Convolutional Network), developed by Dai et al. [15], addressed translational invariance through position-sensitive score maps. R-FCN demonstrated strong performance, achieving an 80.5% mAP on the Pascal VOC dataset with the ResNet101 backbone.

Building on the success of Faster R-CNN, Mask R-CNN extended its capabilities to instance segmentation by adding a RoIAlign layer. Mask R-CNN further improved detection and segmentation accuracy, achieving excellent performance using the ResNeXt-101 backbone.

FPN (Feature Pyramid Network), proposed by Lin et al. [16] in 2017, enhanced object detection by fusing multi-scale features, improving the detection of objects at different scales. FPN delivered state-of-the-art results on the MS COCO [17] dataset, cementing its place in two-stage detectors.

Further innovations in 2018, such as NASNet, used learned features from image classification to improve object detection, achieving a 43.1% mAP on the MS COCO dataset with a Faster R-CNN framework.

In 2020, DETR (Detection Transformer), developed by Facebook AI [18], introduced a transformer-based encoder–decoder architecture for object detection. DETR achieved a 44.9% mAP on the COCO dataset, operating at 10 frames per second.

#### 2.3.2. One-Stage CNN Detectors

One-stage CNN detectors aim to simplify the object detection pipeline by predicting object locations and classes in a single pass, without the need for region proposals. In 2016, SSD (Single Shot MultiBox Detector), proposed by Liu et al. [2], used multi-scale feature maps with a VGG-16 backbone to efficiently detect objects. SSD achieved a remarkable balance between speed and accuracy, operating at 59 fps with its SSD300 architecture.

SqueezeDet, inspired by YOLO and SqueezeNet [19], was designed for real-time embedded applications where power and energy consumption are critical. SqueezeDet achieved 57 fps, making it an ideal choice for real-time scenarios with limited resources, while still maintaining high accuracy.

In 2018, MobileNetV2-SSDLite was introduced by Howard et al. [20] as an improvement for real-time object detection on mobile devices. This model utilized the lightweight MobileNetV2 backbone, reducing the number of parameters and computational load while still providing efficient object detection performance.

In 2019, CenterNet [21], proposed by Zhou et al., advanced key-point-based object detection. By predicting a triplet of key points (center, top-left, and bottom-right) for each object, CenterNet improved accuracy and achieved a 47.0% mAP on the COCO dataset using the HourGlass-104 backbone.

The series of YOLO models achieves the real-time perception for advanced driver assistance systems where low latency and high accuracy are essential for safety-critical applications. In its latest version, YOLOv8, significant improvements guarantee better detection deployment for autonomous driving. With a better CSPDarknet-53 backbone, anchor-free detection, and adaptive feature fusion, YOLOv8 improves vehicle, pedestrian, and traffic sign detection in complex urban areas.

One of YOLOv8’s best improvements for ADAS is its resistance to varying light and weather conditions. Intelligent label assignment and an enhanced loss function enable more reliable detection under challenging conditions such as low light, rain, and occlusions—critical parameters for autonomous driving. The anchor-free methodology also reduces computational requirements, allowing YOLOv8 to run in real time on automotive devices.

Better situational awareness, collision mitigation, and road driver aid allow it to shine as a state-of-the-art object detection architecture for ADAS.

### 2.4. Performance Metrics

In object detection, the performance of these networks is often measured by the mean Average Precision (mAP), which calculates the area under the precision–recall curve. Other key metrics include Intersection Over Union (IoU), which measures the overlap between predicted bounding boxes and ground-truth boxes, and frames per second (FPS), which assesses how fast the model can process images. In Table 1 the fps and AP have been put up for some of the widely used object detection algorithms.

### 2.5. Challenges in Object Detection and Tracking

Object detection and tracking in AVs must handle various challenging conditions, including occlusion, adverse weather, and nighttime.

#### 2.5.1. Handling Occlusion in Tracking

Occlusion is a common challenge in which other objects temporarily block objects. This requires the tracking system to re-identify objects when they reappear. Algorithms like DeepSORT, BoT-SORT, and ByteTrack excel at maintaining object awareness even during occlusion. They use appearance-based and motion-based models to predict object locations.

#### 2.5.2. Adverse Weather Conditions

Object detection becomes more complex in challenging weather conditions like rain or fog [24]. Rain droplets, for example, can obscure critical features. Research on de-raining techniques, including using GANs [25] (Generative Adversarial Networks), has shown that removing rain droplets improves visibility and can sometimes smooth out the image, reducing object detection accuracy as you can see in Table 2. A PReNet [26] was introduced, which iteratively trained a network dedicated to the dense image inpainting using the raindrop mask issued from the semi-supervised stage [26].

Other approaches include domain adaptation, where models trained in clear conditions are adapted to handle adverse conditions using unsupervised learning techniques. A very descriptive paper about the domain adaptation strategy for Faster R-CNN was written in [27].

#### 2.5.3. Nighttime Object Detection

Low-light conditions present another significant challenge for AVs [28]. Nighttime detection suffers from issues like poor illumination and image noise. Recent studies have suggested that incorporating images from dusk and dawn into the training set helps improve the model’s robustness in low-light conditions. In Table 3 is a breakdown of images in the BDD dataset [29]. Refer to the Additionally, hardware solutions such as LiDAR, infrared (IR), and thermal cameras enhance detection performance in the dark.

### 2.6. Datasets for Object Detection and Tracking

Several datasets are widely used for benchmarking object detection and tracking models:

- KITTI Vision: one of the most comprehensive datasets for AVs, providing images and 3D point clouds for object detection and tracking.
- nuScenes: a large-scale dataset for AV research, including LiDAR, camera images, and radar data.
- Waymo Open Dataset: developed by Waymo, it provides extensive data for training object detection and tracking models.
- CityScapes: focuses on semantic segmentation and object detection in urban environments.

Accurate and efficient object tracking is critical to autonomous vehicle (AV) perception systems. It enables AVs to maintain situational awareness, make informed decisions, and ensure the vehicle’s and its surroundings’ safety. Tracking helps AVs monitor moving objects like vehicles, pedestrians, and cyclists across consecutive frames, even when these objects are temporarily occluded. This section explores various object tracking algorithms, from older techniques to state-of-the-art methods, analyzing their strengths, limitations, and use cases, focusing on how they handle occlusion. In Table 4 are few of the most widely used dataset for object detection, tracking and segmentation for AV’s.

**Table 4:** Datasets for autonomous vehicle perception.

Dataset	Real	Location Accuracy	Diversity	Annotation			
				3D	2D	Video	Lane
CamVid [30]	✓	-	Daytime	No	Pixel: 701	✓	2D/2 classes
Kitti [31]	✓	cm	Daytime	80k 3D box	Box: 15k, Pixel: 400	-	No
Cityscapes [32]	✓	-	Daytime, 50 cities	No	Pixel: 25k	-	No
IDD [33]	✓	cm	Various weather, urban and rural roads in India	No	Pixel: 10k	✓	No
Mapillary [34]	✓	Meter	Various weather, day and night, 6 continents	No	Pixel: 25k	-	2D/2 classes
BDD100K [29]	✓	Meter	Various weather, 4 regions in US	No	Box: 100k, Pixel: 10k	-	2D/2 classes
SYNTHIA [35]	-	-	Various weather	Box	Pixel: 213k	No	No
P.F.B. [36]	-	-	Various weather	Box	Pixel: 250k	-	No
ApolloScape [37]	✓	cm	Various weather, daytime, 4 regions in China	3D semantic point, 70k 3D fitted cars	Pixel: 140k	3D/2D video	35 classes
Waymo Open Dataset [38]	✓	cm	Various weather, urban and suburban roads in the US	12 M 3D boxes	Box: 12M, Pixel: 200k	✓	2D/3D lane markings

Note: indicates real dataset and annotation in video available.

#### 2.6.1. Classical Tracking Methods

Before the rise of deep learning, classical tracking methods, such as Kalman filtering and the Hungarian algorithm, were widely used. These methods operate based on mathematical models and object motion assumptions.

- Kalman filtering: This method is commonly used to estimate the future state of an object based on its past trajectory. The Kalman filter is beneficial for tracking objects moving in a linear path with constant velocity. However, it struggles in cases where the object’s movement is erratic or nonlinear.
  Example: In the context of AVs, a Kalman filter might track the position of a moving car on a highway by predicting its future location based on its current speed and direction [39].
- Hungarian algorithm: This algorithm solves the assignment problem—matching detected objects in consecutive frames. It assigns objects from the current frame to the closest objects detected in the next frame, minimizing the overall movement cost. This method can efficiently handle multiple objects but is limited by its reliance on spatial proximity, often failing in complex scenes with significant object overlap [39].

Despite their simplicity, these methods form the foundation of many modern tracking systems. They are computationally efficient but lack the robustness to handle more complex scenarios involving occlusion, appearance changes, or unpredictable object motion.

#### 2.6.2. Deep Learning-Based Tracking

With the advent of deep learning, object tracking has become far more robust and capable of handling complex environments. Deep learning-based tracking algorithms incorporate both appearance-based and motion-based information, allowing them to manage occlusion, re-identify objects, and track through complex interactions. The following discussion explores deep learning-based tracking methods, highlighting their advancements in addressing tracking challenges. In support of this, Table 5 compares the performance of the SORT, DeepSORT, and ByteTrack methods using key tracking metrics.

- SORT (Simple Online and Realtime Tracking): SORT is an early and simple tracking algorithm that uses Kalman filtering for motion prediction and the Hungarian algorithm for object association. It tracks objects solely based on motion models without considering appearance information, which makes it susceptible to errors in crowded environments or occlusion [40].
  Use case: SORT is most effective in environments with minimal occlusion or interaction between objects, such as monitoring traffic in low-density areas.
  Limitation: the algorithm frequently loses track of objects during occlusions due to its sole reliance on motion models, which prevent it from distinguishing between objects based on appearance.
- DeepSORT (Simple Online and Realtime Tracking with Deep Appearance Descriptors): DeepSORT improves upon SORT by incorporating deep appearance descriptors from a convolutional neural network (CNN). This addition helps the algorithm distinguish objects based on their visual characteristics, improving its ability to maintain consistent tracking during occlusion and re-identification when objects reappear after being hidden.
  The improvement over SORT: using appearance-based features, DeepSORT is more robust in crowded scenes or environments where objects frequently overlap.
  Use case: DeepSORT is ideal for dense urban environments or crowded pedestrian areas, where the visual appearance of objects is critical to their accurate tracking.
- Tracktor: Tracktor is a tracking-by-detection algorithm that leverages object detection across multiple frames, eliminating the need for a separate tracking module. Instead, it uses bounding-box regression to predict an object’s future position, making the process more straightforward but dependent on high-quality object detection [41].
  Improvement over DeepSORT: Tracktor simplifies the tracking process by directly extending detection into future frames, though it relies heavily on the quality of the detection.
  Use case: Tracktor performs well in environments where the detection system is highly reliable, such as AVs equipped with advanced LiDAR or radar data for precise detection.
- BoT-SORT (Bytetrack Optimal Transport–SORT): BoT-SORT enhances DeepSORT by incorporating appearance and motion information while using Optimal Transport (OT) to match detected objects across frames. This leads to more accurate tracking, particularly in scenarios with rapid object movement or complex interactions between objects.
  The improvement over Tracktor: BoT-SORT integrates appearance information, allowing it to handle occlusion better than Tracktor, which relies solely on bounding-box predictions.
  Use case: BoT-SORT is especially useful in high-speed tracking scenarios, such as racing or drone footage, where objects move at varying speeds and directions.
- ByteTrack: ByteTrack improves upon tracking by utilizing high-confidence and low-confidence detection. This allows ByteTrack to track objects even when they are partially visible or occluded, reducing missed detection events and increasing overall robustness in challenging environments.
  An improvement over BoT-SORT: ByteTrack’s ability to incorporate low-confidence detection ensures continuous tracking even in scenarios with severe occlusion or partial visibility.
  Use case: ByteTrack is ideal for urban environments, where AVs must track multiple objects under varying conditions, such as dense city traffic or busy intersections.

**Table 5:** Performance scores for SORT, DeepSORT, and ByteTrack. Source: [42].

Metric	SORT	DeepSORT	ByteTrack
MOTA	54.7%	61.4%	77.3%
MOTP	77.5%	79.1%	82.6%
ID switches	831	781	558
MT	34.2%	45.1%	54.7%
ML	24.6%	21.3%	14.9%
FP	7876	5604	3828
FN	26,452	21,796	14,661
Processing speed	143 FPS	61 FPS	171 FPS

#### 2.6.3. Occlusion Handling and Re-Identification

Occlusion occurs when an object is temporarily blocked from view by another object. This is a common challenge for AVs in real-world scenarios, as objects such as pedestrians or other vehicles often move behind obstacles. Handling occlusion requires advanced techniques to ensure tracking continuity and accurate re-identification once objects reappear.

- Appearance descriptors: Algorithms like DeepSORT use visual appearance features to help re-identify objects after they have been occluded. By capturing the unique visual characteristics of objects, these trackers can re-associate objects with their original identities when they reappear.
- Multiple detection strategies: Algorithms such as ByteTrack maintain tracking using high-confidence and low-confidence detection. This ensures that even when an object is partially visible or occluded, its trajectory can still be maintained through lower-confidence predictions.
- Re-identification models: In algorithms like OC-SORT and Tracktor, Re-ID models predict the object’s likely future location based on its previous movements. This helps reassign the object’s identity when it reappears after occlusion, reducing errors in tracking [43].
- Motion modeling and data association: Algorithms employ motion models such as Kalman filters to predict the future trajectory of an object based on its velocity and direction. This allows for consistent tracking even when objects are temporarily occluded.

Each object-tracking algorithm builds upon the limitations of its predecessor, offering improved performance in handling occlusion, re-identification, and object association. While simpler algorithms like SORT and DeepSORT work well in less complex environments, more advanced algorithms like ByteTrack and OC-SORT offer significant advantages in real-world, high-occlusion, and high-interaction environments. These advancements are essential for AVs to ensure safety and efficiency in real-time navigation.

## 3. Localization Strategies in Autonomous Vehicles

Localization is a very crucial part of the operation of autonomous vehicles. The vehicle can know its precise position, orientation, and velocity within the surrounding environment. It is achieved using various sensors and technologies to know exactly where the vehicle is, typically with a centimeter-level accuracy.

The latest advances in precise positioning allow vehicles to localize themselves to a few centimeters and position themselves concerning the road, lanes, and surroundings. This contextual perspective expands the outer horizons of the surroundings concerning the limited immediate view, aiding the vehicle in making intelligent choices like changing lanes, navigating intersections, or formulating a route plan [44]. Localization extends the context so that safety improvements become possible as risks of accidents decrease, and vehicles can anticipate and avoid hazards that have not yet become apparent. It increases safety through sensor fusion by integrating information through different types of sensors (including cameras, radar, and LiDAR) to overcome sensor-specific weaknesses, such as inconsistencies in the data from different sensors that signal possible problems. Localization also helps vehicles navigate other parameters like low light or inclement weather and even GNSS-denied situations [44]. In addition, it functions in conjunction with HD maps, which define the regions of the maps into which the vehicle is placed to guide its actions. It helps vehicles assimilate the position of roads and intersections not instantly within their line of sight, which would aid the vehicles in coping with unanticipated situations or failing sensors. It can be concluded that accurate localization is key to boosting the further development of autonomous driving systems.

### 3.1. Vision-Based Localization

Vision-based localization employs camera images to establish the position and orientation of self-driving vehicles concerning the environment. It analyses the imagery and retrieves particular perspectives in a plane relative to which the motion of the vehicle or its position is ascertained. It is efficient in areas where GPS signals cannot be obtained. It can also be used with other sensors such as IMUs, GNSS, and LiDAR for better performance and more encompassing solutions. Various methods of vision-based localization have shown usefulness in autonomous navigation, including feature-based approaches, visual odometry, and place recognition, among others. Nonetheless, issues such as sensitivity to lighting and texture still remain significant in its usage.

- Feature-based localization: In feature-based localization, differential features from a set of images are used to generate matches between consecutive frames for motion estimation purposes. Such a method is of great importance during such conditions, although chances of encountering such conditions where repetitive textures or low-contrast environments exist are also very high.
- Visual odometry (VO): Visual odometry (VO) concerns the self-location estimation of the mobile robot mounted with cameras or cameras mounted on their platforms [45]. VO has several advantages, such as escaping the GPS fold without compromising on the precision of positioning and being more affordable than other sensor-based systems. It is also less affected by wheel slippage, accommodating rough surfaces, unlike the old-fashioned wheel encoders [46]. However, there are some drawbacks that VO has to face, including the dependence on power resources and the processes of VO being affected by lighting and the environment in general, altering its effectiveness.
- Place recognition: Through place recognition, a person can recall a specific position that he/she has already been to by looking at the picture content. When used with other localization methods, this method can yield good positioning accuracy by enhancing the system’s robustness.
- Integration approaches: vision-based localization techniques usually have additional sensor systems that are used to improve performance.

GNSS/INS integration: combining vision data with inertial measurements can improve the accuracy and availability of positioning solutions.

**Figure 3:**
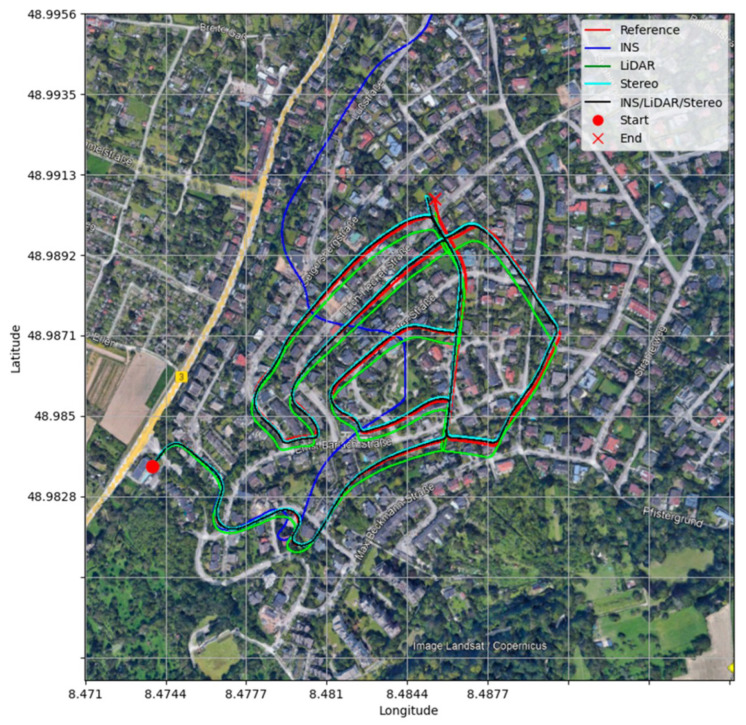
Comparison of trajectories in the world frame (WGS84), D-34, base map captured from Google Earth. Source: [47].

Sensor fusion: Fusing visual data with information from cameras, radar and LiDAR allows more reliable localization, particularly in GNSS-denied areas or challenging environments. Figure 3 shows the comparison of trajectories and the fusion of data from multiple sensors for precision navigation in GNSS-denied environments.

Software-defined networking (SDN)-based smart-city approaches have been implemented recently with success to solve the problems of RNNs-based classification of Non-Line-of-Sight/Line-of-Sight (NLOS/LOS) and GNSS positioning in urban areas. Furthermore, dynamic sensor integration models based on environmental maps and multipath detection techniques have shown improved localization performance across various scenarios.

### 3.2. LiDAR-Based Localization

LiDAR (Light Detection and Ranging) is becoming an important part of the machines mounted on the vehicle for autonomous navigation and has environmental sensing capabilities [48]. LiDAR does this by using a pulsed laser beam and measuring the time taken for the pulse to reflect after hitting a surface. This enables a high density of spatial information represented in the form of three-dimensional point clouds capturing the environment with millimeter precision [48].

Even in LiDAR-based localization, the 3D data described above help to find the accurate placement and direction of a car or robot in the surrounding space. This approach could be used where the conventional GPS would suffer, for example, in urban canyons, indoors, or other places with poor satellite visibility [48]. The localization task is solved by matching the current 3D LiDAR scan with a previously obtained high-resolution map of the area where the vehicle is located or with the map reconstructed along the vehicle track.

Point cloud registration algorithms are the cornerstone technology in localization. LiDAR systems are the principal technique of matching current LiDAR scans with some reference data to understand the vehicle’s position and heading. The purpose of these algorithms is to establish the best possible transformation for the alignment of two point clouds; one becomes the source (or the present scan) and the other the target [49]. One of the most efficient and simple techniques that has been found to be extensively applicable is the Iterative Closest Point algorithm. The ICP algorithm is based on an iterative improvement of point cloud alignments. Each iterative process includes two steps: the first matches point from the source and target cloud, and the second finds a transformation matrix used to rotate the source cloud [44]. This procedure is repeated till either the alignment remains unchanged, or the predetermined iteration number is reached [49].

That being said, ICP suffers from poor performance under initial misalignment, and due to the nature of the solution space in computer vision applications with repetitive structure, it gets stuck in the local optimum. In order to remove those limitations, a sufficient number of ICP variants are proposed as well. These include Point-to-Plane ICP aimed at speeding up the process by reducing the distance from a given point to the tangent plane associated with its nearest point, while Generalized ICP is a more comprehensive system that integrates both point-to-group and point-to-thin-object methods of operation into generalized point cloud matching models. Another variant, Trimmed ICP, improves robustness to outliers by considering only a subset of the best correspondences in each iteration [49].

A different alternative to ICP is the Normal Distributions Transform (NDT), which is a more advanced point cloud registration algorithm. NDT is a particular way of solving this problem in which the surrounding environment is modeled by a set of normal distributions. The point cloud is partitioned into voxels, and mean and covariance are computed for the ten points in a voxel. NDT focuses on optimizing the transformation to maximize the probability of the observed point cloud under the derived distributions. This way of areas is beneficial because even though ICP may be affected by the operational environment, the initial misalignment does not affect the technique’s robustness [49]. The downside is that NDT can be computationally heavy during the first stages as there is a requirement to compute normal distributions for every voxel.

The objective of the present invention is to outline the fundamental features of an approach for developing mobile navigation systems based on a LiDAR sensor framework. One of the main advantages of photogrammetry based on laser scanning is its insensitivity to light changes. In contrast with the systems based on cameras, LiDAR is unaffected by the time of day or the weather (up to a certain severity) and operates even in the dark. Moreover, thanks to the precision of LiDAR, good positioning is also accessible in built-up areas where other sensor approaches struggle with spatial ambiguity with all the relative structures [48].

Nevertheless, LiDAR-based localization also has its disadvantages. For instance, it can be quite challenging to manage the real-time processing of dense point clouds due to their high computational demand, which is why strong algorithms and efficient onboard computing are required. In addition, a structural modification of the environment with an emphasis on partial occlusions or dynamic obstacles needs to be strictly controlled to prevent localization inaccuracies. Thus, a number of researchers focus their efforts on these elements and work on increasing the reliability of LiDAR-based localization in the presence of dynamic factors.

Additionally, important enhancements have been made to systems relying on LiDAR, such as solid-state LiDAR, improved sensor resolution and ranging, pushing the spatial resolution of the LiDAR-enhanced localization systems to a higher level. This approach is also being actively pursued to provide more effective and versatile localization capabilities for self-driving cars and other robotic devices by combining it with other sensors, like cameras and IMUs.

## 4. Mapping Technologies for Autonomous Navigation

**Figure 4:**
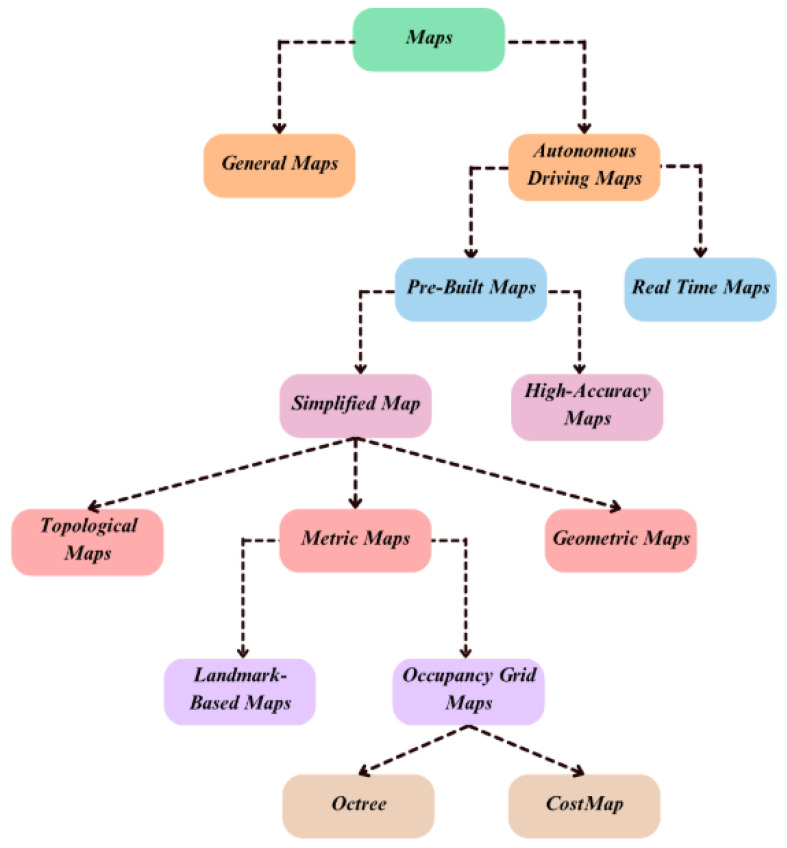
Classification of maps.

Mapping is an important blueprint allowing autonomous vehicles (AVs) to understand, navigate, and react to the environment safely and effectively. High-definition (HD) maps have revolutionized vehicular navigation by moving from traditional GPS systems into comprehensive digital maps or representations of roads and the environment. This procedure involves creating exact and detailed representations of the physical world that the vehicle can utilize in its relocation and navigation decision-making. This survey provides an overview of the mapping module of autonomous vehicles, showing its significance alongside some of the current trends. Figure 4 gives the types of maps used in AV navigation.

### 4.1. What Is a Map?

#### 4.1.1. Definition

In the present-day world, maps are essential in different spheres of life, including navigation, exploration, and geographical comprehension. In navigation, maps are very significant tools used by people and self-driving cars to provide necessary information that enhances orientation and transport [50,51].

#### 4.1.2. General Maps Versus Autonomous Driving Maps

General Maps: General maps provide a broad overview of geographical information, including political boundaries, geographical features, man-made aspects like roads and cities, street networks, points of interest, traffic conditions, and turn-by-turn directions. They assist drivers in identifying the most efficient routes, understanding road layouts, and being aware of traffic restrictions. These maps are designed for a wide audience and are versatile for general navigational purposes guiding humans.

Autonomous driving maps: In contrast, autonomous driving maps transcend their conventional role of guiding human drivers from point A to point B. HD maps are usually employed in autonomous driving and have high accuracy. They enable vehicles to understand their surroundings very well because they provide information about road geometry, lane markings, and signage with centimeter-level precision. Unlike general maps, HD maps stress semantic contents necessary for driving commands; for instance, traffic lights and pedestrian crossings are related to a specific vehicle route on a specific time basis. Geometric information, semantic information, and dynamic updates can be distinguished among components of HD maps. These all-inclusive data are essential for the precise localization and navigation of self-driving cars. The comprehension mechanism is enhanced by HD maps, which help AVs understand their environment accurately, leading to safe and efficient driving decisions.

### 4.2. Types of Maps Used in Autonomous Systems

Maps utilized by autonomous systems serve different purposes, including navigation, localization, and environmental understanding. They vary in complexity, detail, and purpose, meaning each has specific needs in an autonomous system. Every map type performs a distinct function as summarized in Table 6 within autonomous systems, ranging from real-time adaptation to precise localization, efficient path planning, and detailed environmental understanding. They can be categorized as follows:

#### 4.2.1. Real-Time (Online) Maps—Simultaneous Localization and Mapping (SLAM)

SLAM enables vehicles to build maps of their environment while keeping track of their location within these maps. This is particularly crucial in environments where preexisting maps are unavailable or subject to frequent changes. SLAM systems use sensor data from cameras, LiDARs, and range finders to perceive, map, and localize robots in the environment.

#### 4.2.2. Prebuilt Maps

- Simplified map representations: Simplified map representations reduce the computational load while retaining essential navigational features. These maps are categorized primarily into topological, metric, and geometric maps.
  − Topological maps: Topological maps emphasize connectivity and relationships between locations rather than precise geometric details. They simplify high-level route planning and navigation by representing the environment as nodes and edges, highlighting key routes and intersections [52,53]. Topological maps provide abstract representations of paths and landmarks, useful for understanding the overall layout of an area without delving into precise measurements.
  − Metric maps: Metric maps provide detailed spatial information about the environment, including distances and relationships between objects. They are essential for precise navigation and obstacle avoidance in autonomous vehicles.
    ∗ Landmark-based maps: Landmark-based navigation relies on identifying environmental features to determine the vehicle’s position and orientation. They utilize distinct landmarks such as buildings or traffic signs for localization and navigation [54]. These maps are handy in GPS-denied environments [55,56].
    ∗ Occupancy grid maps: These maps represent the environment as a network of cells, indicating each cell’s chances of occupation. They are widely used in determining drivable and non-drivable areas. Occupancy grid maps are crucial for mobile robot perception and navigation, differentiating between free space and obstacles [57].
      · Octree: The hierarchical 3D grid systems are an efficient means of representing three-dimensional spaces. In an eight-node octree, every node has eight children for deeper spatial partitioning. They have additional advantages for extensive three-dimensional regions by enabling spatial data management and processing efficiently [58,59].
      · CostMap: A cost is assigned to each cell in the map, representing the difficulty of traversing different areas. These cell costs guide autonomous systems in choosing the safest and most optimal paths. CostMaps are important in dynamic obstacle avoidance and efficient path planning in autonomous navigation systems.
  − Geometric maps: Geometric maps give accurate geometrical data concerning objects and their locations in the surroundings. Such maps usually use vector data systems for representation, which is important for activities that require high precision, such as avoiding obstacles and navigating accurately through traffic. Thus, geometric maps are employed widely by urban planners to map infrastructure, which includes buildings and roads [60,61].
- High-accuracy maps—HD maps: Autonomous driving needs maps of fine resolution (HD) that yield centimeter-level precision. They contain elaborate representations of road environments, lane configurations, traffic signs as well as obstacles that are regularly updated to reflect real-time situations. Through HD maps, autonomous vehicles can navigate precisely, plan routes, and dodge obstacles, relying on various sensors like LIDARs, cameras, or GPS for their creation.

### 4.3. HD Maps

**Figure 5:**
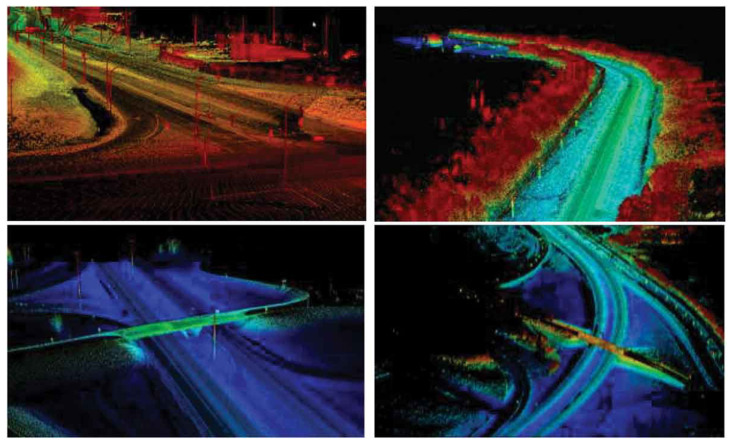
LiDAR point cloud maps depicting road shapes and stationary objects. Source: [70].

Unlike traditional digital maps, suitable for most activities, AVs work at a different level and cannot employ regular maps. This is simply because they do not contain detailed information about the traffic lights and their locations, traffic lanes, the height of traffic light poles, and even the accurate bending of roads. High-definition (HD) maps are essential for self-driving automobiles because they are three-dimensional maps composed of precise measurements of the surroundings to the centimeter level in all directions and accurate positioning [62,63,64]. These detailed HD maps are unlike simple standard maps because they do not use such basic maps for autonomous vehicle navigation in their operation. These maps are neither outlined in the course of an experiment nor are they a one-off generic course; they are strategically outlined and repeated consistently throughout the experiment. These digital maps come in a very large detail and accuracy [65,66] to give appropriate context and perspectives, especially in areas where GPS signals are weak, such as urban canyons and tunnels, which are important for effective navigation. They act as an improved perception system of the vehicle by including prior information such as road shape, lane markings, and stationary objects, which is beneficial especially when the weather is not clear such as during snow or fog as illustrated in Figure 5. For example, looking through a camera in dense fog, which greatly limits visibility, an AV can utilize HD maps with the help of several sensors and navigate using trusted data of the surroundings. To work as expected, HD maps most often incorporate some localization system that is usually GNSS with RTK correction [67,68,69]. This device enables the vehicle to position itself in real time using the map. However, dynamic elements such as other vehicles or even pedestrians as well as construction sites cannot be represented in the static mapped data; hence, a strong perception engine is necessary for tracking changes in real time.

Additionally, high-definition maps include real-time information about traffic patterns, construction activity, and temporary road closures so that autonomous vehicles can cope with environmental dynamics. HD maps are primarily created offline; however, once created, they need to be properly maintained through real-time updates for relevance and realistic utilization.

HD map creation is a complicated and multi-layered process requiring the convergence of technology, processes, and people. To improve the quality and trustworthiness of these maps, a human-in-loop technique that utilizes the best of both worlds, AI and humans, has been suggested. Such a scheme is crucial for predicting the path of vehicles likely to operate in real-time conditions with unusual or borderline events.

Especially during the last few years, there have been many recent HD maps generated and subsequently maintained, caused by the overwhelming growth of sensors, computational resources, and machine learning methods. The advancements have simplified data gathering, enhanced mapping precision, and suggested a new effective way of updating the maps that creates opportunities for effective and efficient HD mapping that is more dependable and scalable.

Despite the fact that HD maps are very important, it is worth mentioning that in the field of autonomous driving, there does not exist a unique standard structure for an HD map. HD map design structures and processes vary from one organization to the other, stressing the absence of a single methodology.

#### 4.3.1. Key Features of HD Maps

Compared to the typical mapping tools, HD maps allow for easier and more accurate navigation since they have more details, including the following ones, which are built into the map:

- Painted lines: cast and represented as defined 3D features that border a particular roadway.
- Traffic signs: these are strategically placed for easy identification and reaction.
- Three-dimensional building models: implemented to give a better view of cities and their spatial organization.
- Signals and stop lines: these two are angles and 3D spaces related to stopping and other actions at the intersection.
- Semantic data: encoding information concerning the driving features like lanes, junctions, and the different road segments into the maps for advanced driving support functionalities.

The ability to achieve such a level of detail is fundamental for AVs as they need to understand their environment to within even a few centimeters in order to move safely.

#### 4.3.2. Main Categories of Mapping Information

In highly automated driving systems, high-definition maps contain several layers of information to aid in navigation and decision making. Below are the main types of mapping information.

- Topological representation
  − What does it denote?
    In the context of autonomous vehicles, a topological map is used to represent the connectivity of key elements—such as roads, junctions, traffic signals, and lane markings—without requiring exact geometric details. This abstraction enables efficient path planning by focusing on how these critical nodes are interconnected rather than their precise spatial positions [52,53].
  − Why is it necessary?
    This enables the system to appreciate how different regions are connected and related, which is important in route planning and navigation. For example, a topological view of a road allows the system to find its way even when other factors are present, such as traffic or road reconditioning, allowing it to calculate other routes [71].
  − Use case:
    It can provide other routes to take when there is heavy traffic.
- Geometric representation
  − What does it denote?
    The configuration and relative positions of road surfaces, buildings, lanes, and other infrastructures [62].
  − Why is it necessary?
    Autonomous systems must deal with geometric details regarding localization, route design, and movement. The vector data structure is most used where real-life objects are reduced to points, lines, polygons, and so on.
  − Use case:
    Geometric representations come in handy for specific movements whenever an autonomous vehicle has to position itself in a particular lane or steer clear of curbs and other objects.
- Semantic interpretation
  − What does it denote?
    It adds meaning and gives purpose to the spatial-structural and –topological aspects of roads by attaching names to roads, their tributaries, road signs, pedestrian crossings, limits of speed, etc.
  − Why is it necessary?
    The meaning of the semantic information is to make machines capable of understanding and perceiving the environment with the help of navigation. This is essential when decisions have to be made based on certain principles and the state of street traffic [54,72,73]. Examples: the HD map layers provided by HERE [74] and TomTom’s RoadDNA [75] contain semantic features at the object level for the vehicle’s positioning and enhancement of the decision-making process.
  − Use case:
    The system identifies a traffic sign and performs related operations.
- Dynamic elements
  − What does it denote?
    Elements that are based outside someone’s control, such as moving people and vehicles, items that are present on the area surrounding the road, as well as changes concerning the road that may be temporary, such as construction work and traffic congestion.
  − Why is it necessary?
    In such environments, objects are permanently positioned and in motion simultaneously, so the elements of autonomous systems must be able to interact with them instantly for navigation to be safe. The changes are timely and precise; therefore, they ensure that the HD map is current and appropriately describes the surroundings.
  − Use case:
    The vehicle changes its orientation and moves around children, adults, and other cyclists to avoid knockdowns.
- Feature-based map layers
  − What does it denote?
    These are comprised of several map layers that are rich in specific elements in the map that favor the process of localization and navigation [76].
  − Why is it necessary?
    These layers also increase the maps’ precision and trustworthiness, thus aiding the system in operating effectively in environments with many structures, such as cities where outdoor navigation is challenging due to poor satellite reception [77].
  − Use case:
    In an urban setup surrounded by tall structures, feature-based map layers assist the vehicle in steering accurately by using moving objects such as traffic lights and similar features that are easily identifiable.

**Figure 6:**
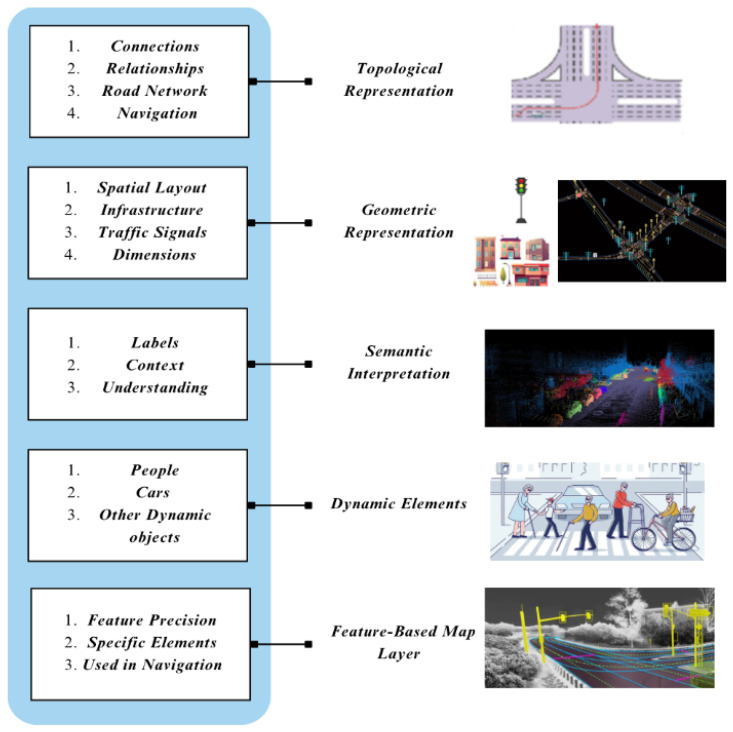
Main components of HD maps.

The layers include topology, structure, semantics, dynamics, and features, variously referred to as dimensional HD maps, which are essential for the overall working of any autonomous driver system (Figure 6). Each dimension plays an important role on its own regarding operation, navigation technology, and safety.

#### 4.3.3. HD Map Creation Pipeline

Creating high-definition maps for autonomous driving requires several steps to go from raw collected data to an exact and extensive map for navigation. Creating HD maps is a very difficult and stepwise task that involves various tasks such as collecting data, preparing data, sensor fusion, point cloud aligning, deducing certain features, and map making. Scenario descriptions play a pivotal role in the framing of perception and mapping tasks to autonomous vehicles. Scholtes et al. (2021) proposed a layered model with six layers for urban traffic categorization, for structuring HD map generation [78]. Similarly, Stepanyants and Romanov (2022) presented an object-oriented pattern for structuring interaction between traffic participants and machine perception in driving scenarios [79]. Both works provide the methodology for organizing the HD map creation pipeline, data collection, and processing, corresponding to structured urban traffic representations. For ease of understanding, we can categorize the entire process into four components: data collection, data preprocessing and alignment, map making, map updating, and feature extraction.

**Figure 8:**
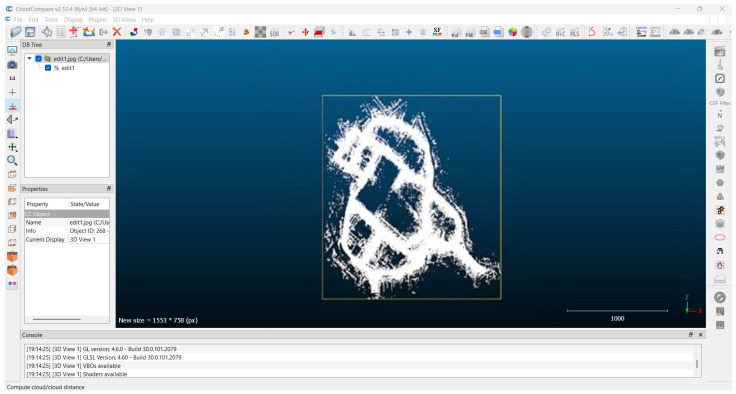
Preprocessing of point cloud data.

- Data collection:
  The first stage is collecting the needed data to develop an HD map. A vehicle that has been installed with high-accuracy sensors and improved calibration techniques is sent to map out and gather extensive details of the surroundings. Mapping vehicles are usually fitted with Mobile Mapping Systems (MMSs) [80,81], which consist of various sensors most of the time. Figure 7 shows the Mobile Mapping System in use with relevant sensors.
  For instance, the LiDAR, the GNSS, the IMU, and the camera of an MMS user work together to deliver a dense and accurate three-dimensional point cloud of the scanned scene. Cameras are used to photograph the environment to produce high-resolution images with details like road markings, traffic signs, and features. Similarly, another type of sensor which is found in the MMS is the Global Navigation Satellite System (GNSS). GNSSs have receivers that allow them to connect with several satellite systems simultaneously, improving the accuracy of the measurements. A GNSS sensor is frequently integrated with the Inertial Measurement Unit (IMU) sensor to compute the moving object’s trajectory, including its position, velocity, and orientation in space [82,83].
  Once the data are collected, they are processed through a series of steps to generate an HD map. The data collection process must ensure the integrity and quality of the data to achieve accurate HD maps.
  **Figure 7:**
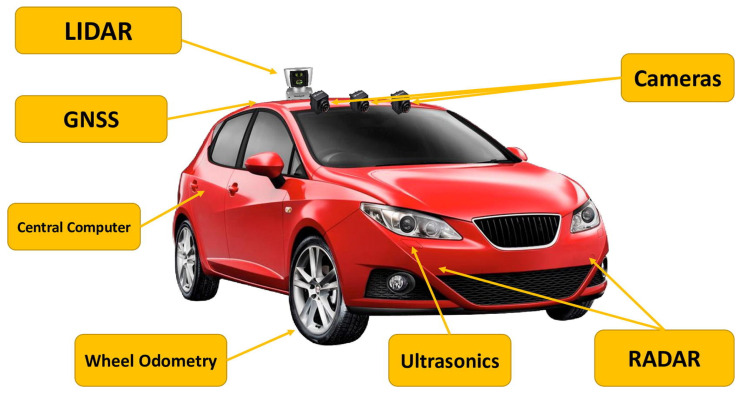
Mobile Mapping System. Source: [84].
- Data Preprocessing and Alignment:
  Data preprocessing and alignment are extremely important factors in HD map generation as they increase the collected data’s accuracy, consistency, and quality. Data preprocessing is manipulating gathered data from different types of sensors by cleaning, filtering, and sieving them to remove unwanted signals, outliers, and inconsistencies. This is meant to ensure the information collected is as good as possible and fit for the subsequent processes. Figure 8 shows the use of CloudCompare v2.12.4 software [85] to preprocess point cloud data by integrating point cloud segments and applying SOR filtering to remove noise and enhance data accuracy. This procedure usually consists of sensor calibration, synchronization of time, and the “registration” of spatial coordinates [86]. It is very important to carry out calibration in order to obtain precise readings and position the sensor data properly. It helps when combining scanner data collected by synchronizing time across different sensors. It is the last stage where the data are shaped to fit into a common coordinate frame for proper positioning of the sensor data.
- Feature extraction:
  Feature extraction is an essential part of the HD map creation pipeline, wherein relevant data are identified and extracted from the processed material. Feature extraction entails spotting and drawing out key features from the aligned data, such as the edges of the road, lane markings, road signs, and other pertinent objects. Numerous methods can be employed for the purpose of feature extraction, for instance:
  − LiDAR feature extraction: a LiDAR sensor captures a very dense 3D cloud of points, which is then processed to extract features like road edges, curbs, and obstacles whose features can be identified and extracted using techniques such as clustering, segmentation, and other machine learning algorithms [87,88,89,90].
  − Image feature extraction: cameras produce high-definition stills that can be analyzed using computer vision techniques to extract features like lane markings, traffic signs, and road surfaces [91,92].
  Sensor fusion techniques improve the reliability of feature extraction by combining data from LiDAR and cameras rather than using one sensor only [93,94,95,96].
- Map creation and updating:
  The last phase of the HD map building process consists of the map’s enhancement or editing while using the detected characteristics. This phase usually concerns the following:
  − Map data structure—the detected features have to be transformed into a proper representation so that they can be efficiently stored, accessed, and worked upon (for example, a graph or a tree) [97].
  − Map annotation—the labeling of the various extracted features, their types such as lanes, speed limits, and even traffic sign attributes to enhance and enrich the hyper-detailed maps [97,98,99].
  − Map validation—the process of checking the quality and coherence of the HD map content against the reference data, for example, collected surveys or other maps. Different methods may be utilized within the validation process, such as an inspection which can be performed visually or statistically, or by using deduced techniques [100].
  − Map updating—Modifying the HD map to include any evolutions experienced within its surroundings, for example, new roads, lane re-configurations, or even the presence of temporary obstacles. This also includes real-time data alterations regarding the constantly changing circumstances and location within the existing local map [101,102].

#### 4.3.4. Advantages of HD Maps

- Correct localization: Ensuring the proper positioning of a vehicle in urban areas where GPS is unreliable becomes simple due to the use of HD maps, which are accurate down to centimeters [55,103,104].
- Better route design: HD maps provide information concerning the design and configuration of the structure of roads, including lanes and traffic regulation, making it possible and less risky to plan out routes.
- Better awareness: autonomous vehicles receive more sensor-rich information about the geographical area via the use of HD maps, hence better perception of the vehicles is achieved even in extreme weather and visibility conditions [105,106].

#### 4.3.5. Limitation of HD Maps

**Figure 9:**
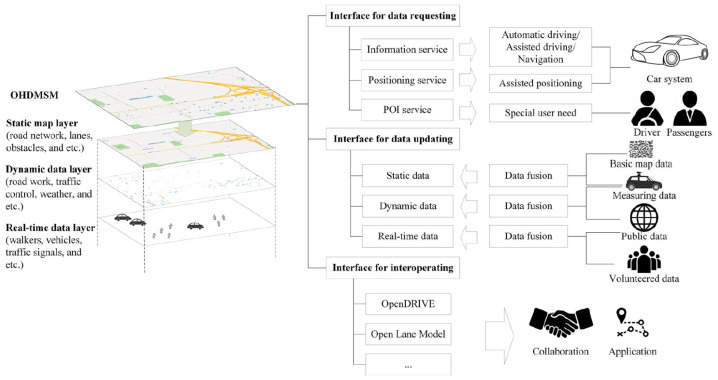
Open HD Map Service Model. Source: [109].

- High cost of creation and maintenance: Creating and maintaining high-definition maps is resource-heavy [107]. This involves an accurate mapping of a city or a region over years and enormous amounts of money. In addition, real-life situations are always changing, so the maps have to be frequently and expensively corrected [101,108]. Various methods are incorporated to update the map constantly, one being the SLAMCU algorithm, which effectively detects and updates high-definition map changes, improving autonomous driving by providing the most accurate environmental information [102].
- Standardization: The absence of common formats and protocols for HD maps reduces the levels of sharing and cooperation between the different players in the autonomous driving value chain [107]. Now similar to Open HD Map Service Model (OHDMSM) (Figure 9 shows the OHDMSM framework), attempts are being made to create interoperable HD map data models, a baseline that would assist in HD mapped development and unify data fusion and applications across platforms [109].
- Data storage: HD maps are also quite large, involving several terabytes of digital storage space whenever large city-based map coverage is needed [110]. This presents a problem to self-driving cars as storing the whole map at once may not be possible. Rather, the vehicle can connect to the internet, which allows for the maps to be continuously uploaded and downloaded on the fly. However, there are disadvantages to this approach, such as the need for considerable bandwidth, the risk of congestion, or even unavailability of the networks in areas that are poorly connected [111]. Due to the above reasons, there are heavy cost implications and technical difficulties in using HD maps on a sustained basis.
- Third-party dependency: HD maps are normally made available by a handful of specialized vendors, which makes it impossible to avoid reliance on those third parties concerning map generation, modification, and maintenance. This dependence can further restrict the flexibility and adaptability of the autonomously driven systems since the success of those systems depends on how effective and fast the respective vendors’ systems are updated [112].
- Cybersecurity risks: all systems and applications integrated into the autonomous vehicle must regularly receive updates of the high-definition maps system, thus requiring open communication ports that subject the systems to cyber-risks [112,113,114,115].
- Inapplicability to off-road use cases: HD maps assist in navigating moderate traffic and urban public roads. However, they are not very useful for extreme unstructured environments, such as in mining or agriculture or even in rural areas devoid of paved roads. Most often, in such surroundings, the accuracy of the HD map is either not required or is meaningless.

### 4.4. The Mapless Approach: A New Way Forward?

Another growing trend that does away with the need for HD maps is using real-time perception systems for navigation [116]. Some companies such as Imagery, for example, support a mapless method [117] whereby onboard cameras and sensors are used to survey the vehicle’s surroundings, creating a live environment model. Such a solution has the following merits:

- Flexibility: Cars do not have to stick to the already mapped roads only and can freely move around unknown areas based on inputs from the sensors in real time [118,119,120].
- Reduced costs: this removes the building process and modifying expensive data [121].
- Current information: the operation is present, therefore allowing the system to cope with situations like rerouting [122] or an obstruction on the roads, etc.

### 4.5. Key Technologies for Mapless Approach

- SLAM (Simultaneous Localization and Mapping): it has also been implemented in various mapless navigation systems to create and renew the environment maps while locating the vehicle simultaneously [118].
- Deep reinforcement learning: This is the most commonly used form of deep reinforcement learning employed for training navigation policies, which enable high-rated navigation without collision using local sensors, achieving major improvements in motion planning [119,120].

This mapless approach is based on SLAM, discussed in the Localization Section.

## 5. Simultaneous Localization and Mapping (SLAM) for Navigation

While LiDAR-based localization excels in known environments, it faces limitations when preexisting map data are unavailable or outdated. This challenge gives rise to Simultaneous Localization and Mapping (SLAM), a more comprehensive approach to navigation.

Simultaneous Localization and Mapping (SLAM) is a crucial component of autonomous driving systems, enabling vehicles to simultaneously navigate and build maps of their surroundings. This technology has revolutionized the field of autonomous vehicles by allowing them to operate in real-world environments without relying solely on pre-programmed routes or external positioning systems. SLAM extends localization techniques by determining the vehicle’s position and constructing and updating environmental maps in real time. This dual functionality allows autonomous vehicles to navigate unknown areas, adapt to dynamic environments, and maintain accurate positioning without external references like GPS.

**Figure 10:**
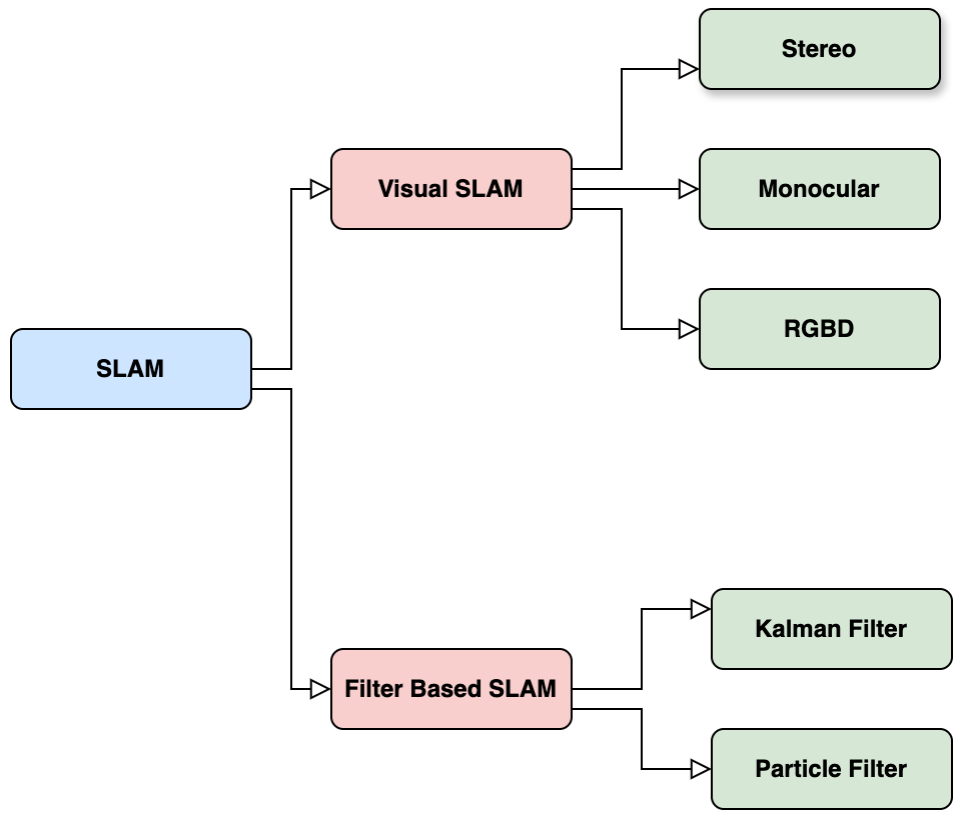
Classification of various SLAM techniques.

In LiDAR-based systems, SLAM algorithms build upon point cloud registration techniques used in localization, adding the complexity of map building and updating. As the vehicle moves, new LiDAR scans are used to estimate position and refine the map, involving complex data association to determine whether observed features are new or previously mapped. Combining LiDAR’s high-precision sensing with sophisticated SLAM algorithms, autonomous vehicles achieve environmental awareness and adaptability crucial for safe real-world operation. This technology enables navigation in diverse environments, from urban streets to off-road terrains. The field of SLAM encompasses various approaches, including filter-based methods, graph-based optimization, and multi-sensor integration, all aimed at enhancing the accuracy and reliability of simultaneous localization and mapping systems. Figure 10 below highlights the different SLAM techniques discussed.

### 5.1. Visual SLAM

Visual Simultaneous Localization and Mapping is a critical technology in autonomous driving. It involves estimating the position and orientation of the vehicle with respect to its environment and generating a map of that environment simultaneously. There have been many approaches to handling this problem, and visual-based SLAM is one of them. The main goal of vSLAM is to predict the camera’s trajectory while reconstructing the surrounding environment. Here, the primary sensor used is the camera because of its availability and ease of sensor configuration [123].

Monocular cameras are the first choice when it comes to visual SLAM approaches. The initial approaches proposed by [124,125] led to a lot of unwanted computations, as each frame was processed to estimate the locations of the map and the pose of the camera as there was little to no information change in those consecutive frames. To solve this, keyframe-based approaches were introduced, and only selected frames that were different from each other were processed. One of the most popular approaches for keyframes was PTAM [126], in which camera tracking and mapping were split into parallel threads to reduce compute complexity, but it had a couple of problems. Firstly, it was still limited to smaller environments, and its performance could degrade when scaled to larger, complex environments. Secondly, PATM did not inherently handle loop closures.

To solve this, [127] came up with ORB-SLAM, which, firstly, uses Oriented FAST and Rotated BRIEF features that are fast to compute, hence helping in complex and large environments. Secondly, it also recognizes previously visited locations and solves the loop closure problem that plagued PATM. Thirdly, it uses a global map with local features for better performance, unlike PATM, which focuses more on the local map, which sometimes causes a drift during the mapping process.

Monocular cameras cannot obtain depth information as they are just 2D projections of the 3D environment; hence, stereo cameras are used. Several approaches have been implemented using stereo cameras. ProSLAM was proposed by [128] as a lightweight and modular stereo visual SLAM mechanism. Similarly to ORB-SLAM, it also used feature matching techniques for keypoint detection and description, such as the FAST algorithm. It was tailored for RGBD AND perspective stereo camera systems. The authors of [129] proposed a highly scalable and proper vSLAM technique called OpenVSLAM, which used rolling shutter cameras in smartphones. This approach was as good as the previous ones, with one significant edge over the others: it had the ability to load and store map databases, which could be used to run localization on prebuilt maps, which is crucial for real-time applications. Regarding direct methods, LSD-SLAM is one of the more popular methods of VSLAM; this method does not involve feature extraction for keypoint detection; rather, it uses the intensity information of each pixel from the image data directly to reconstruct the map and estimate the camera pose. Currently, ORB-SLAM3 [130] is the best method for vSLAM, whether using monocular, stereo, or RGBD camera configurations.

As presented in [131], the 90° stereo vision approach likely offers superior depth-sensing capabilities and potentially more detailed maps compared to the monocular 90° view. However, because of the need for stereo processing, it may come with increased computational complexity and higher hardware requirements. In contrast, the monocular 90° view is generally simpler and less resource-intensive but may struggle with accurate depth estimation and occlusion handling.

### 5.2. Filter-Based SLAM

Filter-based SLAM is one of the most popular techniques when it comes to SLAM. It uses probabilistic methods to estimate the robot’s pose and map the environment simultaneously. The main merit of using these methods over vSLAM ones is the significant reduction in computing requirements and more straightforward implementation. As this method uses probability functions, it is more inclusive of outliers than vSLAM. Let us discuss the methodology behind filter-based SLAM:

- Initialization step: The state vector consists of the robot’s pose (position and orientation) and the environment map. Initially, the state vector is initialized with [x0,y0,theta0], which are the starting coordinates; if they are unknown, it is initialized as [0,0,0]. If there are any landmarks, they can also be initialized in the state vector as x = [x0,y0,thta0,l1x,l1y,…]. The covariance matrix, which is used to consider the uncertainty in the robot’s pose, is also initialized. The diagonal elements of the covariance matrix indicate how certain the initial estimates are.
- Prediction step: Odometry data are used as control inputs for predicting the next state. A motion model describes the robot’s movement given the above parameters. The current state of the robot and the control inputs are passed as inputs to the model to calculate the predicted state. The covariance matrix is also updated to reflect the change in uncertainty due to the robot’s motion. The Jacobian $F_{t}$ of the motion model is calculated to linearize the model around the current state. The updated covariance matrix is
  (1) $$P_{t|t-1}=F_{t}P_{t-1}F_{t}^{T}+Q_{t}$$
- Correction step: The sensor measurements are incorporated to correct the predicted state. These measurements are obtained using a number of sensors like a camera, LiDAR, IMU, etc. These measurements provide information about the environment and the robot’s interaction with it. A measurement model is used to obtain the expected values from the predicted state. The Jacobian of the measurement model is calculated to linearize it with respect to the state vector to linearize the model. This, in turn, is used to calculate the Kalman gain, which is used to correct the predicted state based on the difference between the actual and predicted parameters. The covariance matrix has also been updated accordingly to reflect these changes.

### 5.3. Kalman Filter and Its Variations

The Kalman filter uses a linear estimation to produce the Kalman gain. The dynamic motion and measurement model are used to come up with an a posteriori estimate, which is used to update state estimates and minimize error. This is used in practice in many approaches. Ref. [132] mentioned a scenario in which a one-DoF robot maintained a straight path with a velocity while sensing stationary landmarks. A linear Kalman filter was used to estimate the robot velocity and landmark positions. Here, the linear KF algorithm worked flawlessly. The problem with the linear Kalman filter is that it only works well with linear observation and motion models, which restricts its practical applicability.

To solve this, the extended KF was introduced to handle nonlinear motion and observation models by linearizing them using a Jacobian matrix, making it more applicable in more realistic scenarios. In [133], hardware and software implementations were used to check the applicability of EKF. In the software implementation, a Python program was developed to predict the landmark locations and the estimated track using wheel odometry and laser scans as input; this was tested in a simulation. In these tests, the software implementation gave almost perfect results, whereas the hardware implementation faced quite a lot of challenges as there was significantly more noise in the real-life scenarios.

To optimize this even further, an unscented Kalman filter is used. Unlike the EKF, it does not use linear approximations. Instead, it uses a deterministic sampling approach to capture the accurate probability distribution of the state. It avoids the use of the Jacobian matrix, greatly reducing computational complexity. It captures the higher-order values of the Taylor expansion series and provides a more accurate representation of nonlinear systems. Due to its nonlinear approach, it makes it less sensitive and more robust against model errors.

#### 5.3.1. Comparison and Applications

Comparative studies have shown that the UKF generally provides more accurate representations of state distributions than the EKF [134], especially for highly nonlinear systems. This is evident in experiments comparing the EKF and UKF performance in autonomous navigation setups.

In practice, the choice between EKF and UKF depends on the specific application:

- The EKF is simpler to implement and computationally efficient for mildly nonlinear systems.
- The UKF offers superior performance for highly nonlinear systems, potentially providing more accurate uncertainty representation.

Both filters are widely used in robotics and autonomous vehicle applications, with ongoing research focusing on improving their performance and efficiency

#### 5.3.2. Hardware Implementation Challenges

While software implementations of the EKF and UKF often yield promising results, hardware implementations face significant challenges:

- Real-world environments introduce more noise and variability than simulated scenarios.
- Sensor noise and limited precision can affect the accuracy of state estimates.
- The computational requirements of the UKF can be higher, potentially limiting real-time performance on resource-constrained hardware.

Ongoing research aims to develop more robust hardware implementations that effectively utilize advanced filtering techniques like the UKF in real-world conditions.

### 5.4. Particle Filter and Its Variations

Particle filters, referred to as Sequential Monte Carlo methods, are regarded as an effective method for resolving the Simultaneous Localization and Mapping (SLAM) challenge in a robotic field. Fundamentally, these filters use a weighted particle set to capture information on uncertainty about the robot’s position and the characteristics of its surroundings. Every particle may be regarded as a hypothesis for the robot’s state and a tentative map of the surrounding environment, with a weight representing the probability of correctness of that hypothesis.

As implemented at the basic level in the SLAM particle filter [135], there is a cyclic process consisting of a prediction and an update. Consider particle prediction first; in this step, the particles are displaced in accordance with a motion model of the robot’s behavior. The weights of these particles are modified in response to sensor data during the update phase, whereby each weight modification is performed with respect to the observed weight predictions of each particle. Then comes the resampling phase, where there is a replacement of the copies of the low-weighted particles with the higher-weighted ones to concentrate computational power on the most likely hypotheses.

FastSLAM [136] is one such improvement showing a difference from the other methods. It allows the SLAM to be decomposed in terms of the pose and position only, while using all available measurements. This enables every particle to estimate only one robot pose and maintain several Kalman filters for the uttered landmarks, and this guarantees the high efficacy of the process for the growing map size. Therefore, after further development, FastSLAM version 2.0 was introduced [137], where the proposal distribution was improved with the insertion of current measurements, leading to reduced errors in the particles and thus resulting in a more accurate model even in regions of low features.

The emergence of this area of research has brought new trends in systems dealing with computational load, including several variations of the underlying principles. In this method, the computational burden is carried out by several processors in the networks, which makes the method suitable for large-area scenarios; however, there are still issues, such as synchronization and even particle exchange. Last but certainly not least is adaptive particle filter SLAM [138], which adapts to the current situation as it varies.

Many parameters must be considered during the realization of particle filter SLAM systems. The number of particles used is a common attention point, which is a trade-off regarding precision versus computational burden. The active choice of the algorithms performing resampling may lead to significant improvements, especially with regard to the problem of particle deprivation—situations when there is no longer any diversity in the set of particles. Accurate sensor models are required for appropriate measurement likelihood; a robust loop closure detection is still necessary to guarantee consistent mapping over time.

## 6. Challenges

The deployment of autonomous vehicles (AVs) in real-world environments presents several challenges that impact perception, localization, and mapping. While advancements in deep learning, sensor fusion, and mapping techniques have significantly improved AV performance, achieving full autonomy remains a complex task due to numerous obstacles. These challenges arise from the dynamic and unpredictable nature of road environments, sensor limitations, high computational demands, and ethical concerns.

To systematically identify and analyze these challenges, we conducted an extensive review of the existing literature, experimental evaluations, and real-world case studies. Our approach involved examining the limitations of state-of-the-art object detection, tracking, and localization techniques, as well as analyzing dataset biases and the impact of environmental conditions on AV performance. Additionally, we assessed computational constraints in real-time processing, and we considered the difficulties in integrating multiple sensor technologies and the scalability challenges in adapting AV systems to diverse geographical and infrastructural settings.

The following points outline the key challenges that hinder the widespread adoption and operational reliability of AVs. Each challenge is discussed in detail, highlighting its effects on perception, localization, and mapping.

- Dynamism and unpredictability: in an ideal world, all roads would be wide enough to accommodate intentionally designed cars that automatically transport passengers; we would need to consider scenarios where there are construction sites, other vehicles, or even pedestrians that do not interact directly with the car sensors; therefore, to receive uninterrupted traffic data, extensive autonomous navigation would have to be put into practice.
- Weather and lighting conditions: Autonomous systems can receive more accurate data in optimal conditions, although this is not always the case, whether we are looking at weather conditions such as rain, snow, or general overcast skies or the time of day reducing sensor efficiency. Even with models such as GAN or thermal sensors being developed, the weather is still critical to automatic navigation.
- Occlusion and re-identification: Having to manually follow certain features across frames for tracking is strenuous, especially in the case of occlusion, whereby one or multiple tracked objects are surrounded by other objects. Although specific models such as DeepSORT or ByteTrack are effective for operational environments with fewer people, they are not mainly designed for complex environments.
- High computational demands: High standards such as real-time output when performing a variety of tasks, including identifying and mapping objects from three-dimensional sensors, are now theoretically possible. However, this would require spending a large sum of money on advanced hardware, making it inaccurate in predicting real-life scenarios.
- Data bias: Datasets such as KITTI, nuScenes, and Waymo Open Dataset are being used to train autonomous vehicle models. Although these datasets are pretty broad, they may also be limited in geographical coverage, weather conditions, and social culture, which can hinder the model, especially in places where such conditions exist.
- Cybersecurity risks: The fact that AVs are constantly communicating with each other, transmitting updates of maps and feedback of sensors, makes them more prone to cyber-attacks. Navigation, privacy, and even vehicle collision can be controlled by hacked devices, which is why robust systems are required to maintain security.
- Fusion of different technologies: Integrating AI technologies such as cameras, LiDAR, radar, and GNSS is challenging. Each sensor comes with its own set of limitations, and amalgamating their outputs to form a single system requires sophisticated algorithms for sensor fusion that are both computationally efficient and robust.
- Legal and social issues: Laws have not been made to adapt to the advancements in technology, and this causes a lack of safety standards and regulations in case a vehicle ends up being involved in an accident. Ethical problems arise when a decision needs to be made in an unavoidable accident, which makes the integration of AVs more difficult.
- Scalability and deployment in untouched or remote areas: Existing AV technologies are built around large cities and flirt with the suburbs with amenities such as lane markings and HD maps. Adapting such systems to rural or cross-country operations is still an uphill task due to sparse data availability and weak sensors in unpaved areas.

## 7. Discussion and Future Scope

As autonomous vehicle (AV) technology advances, several key challenges and opportunities shape its development. While significant progress has been made in perception, localization, and mapping, real-world deployment remains hindered by sensor limitations, computational demands, data biases, and cybersecurity concerns. This study evaluated various methodologies to determine their effectiveness in addressing these challenges, revealing both strengths and areas for improvement.

One major finding is that deep learning-based perception models, such as YOLOv8, Faster R-CNN, and SSD, perform well in structured environments but struggle in complex scenarios involving occlusion, low lighting, and adverse weather conditions. While tracking algorithms like ByteTrack and BoT-SORT demonstrate strong object re-identification capabilities, their performance declines in dense and unpredictable traffic conditions. These results emphasize the need for enhanced sensor fusion techniques that integrate data from LiDAR, radar, and cameras to improve object detection accuracy under varying environmental conditions.

In localization and mapping, our results confirm that LiDAR-SLAM and HD mapping provide superior accuracy compared to camera-based methods, particularly in GPS-denied environments. However, a critical limitation observed was the reliance on preexisting HD maps, which can quickly become outdated in dynamic urban settings. SLAM-based mapless navigation, leveraging real-time updates, emerges as a promising alternative, but its success depends on loop closure detection, real-time optimization, and computational efficiency. To enhance scalability, sensor fusion combining LiDAR, IMU, and GNSS proves to be the most reliable localization strategy, balancing accuracy with adaptability.

A notable challenge in AV adoption is dataset diversity. Current training datasets, including KITTI, nuScenes, and Waymo Open Dataset, exhibit geographic and environmental biases, limiting their generalization to rural, extreme weather, and culturally different driving conditions. Expanding dataset coverage and incorporating synthetic data generation through Generative Adversarial Networks (GANs) can improve AV adaptability across diverse scenarios.

Another critical area of concern is computational efficiency. High-performing models often require significant processing power, making real-time deployment challenging, especially for cost-sensitive applications. The integration of 5G and edge computing can alleviate onboard processing burdens by offloading computational tasks, enabling faster decision-making while reducing hardware constraints. However, as connectivity increases, cybersecurity and data privacy risks must be addressed to prevent threats such as data breaches, GPS spoofing, and man-in-the-middle attacks. Strengthening data encryption and multi-signature authentication protocols is crucial for ensuring secure AV communications.

Beyond technological advancements, the ethical, regulatory, and societal aspects of AV deployment must be considered. Establishing industry-wide safety regulations, liability frameworks, and ethical decision-making models will be essential for public trust and large-scale adoption. Additionally, AV technology holds potential beyond urban mobility, with applications in agriculture, mining, and logistics, where automated off-highway systems can enhance efficiency and safety.

Overall, our findings highlight the importance of adaptive AI models, robust sensor fusion, real-time SLAM, and secure AV networks in shaping the next generation of autonomous navigation systems. Addressing these challenges while leveraging emerging technologies will ensure that AVs are not only scalable and efficient but also capable of navigating safely in complex, real-world environments.

## 8. Conclusions

Object detection, tracking, localization, and mapping techniques are continuously evolving, leading to augmented performance in autonomous navigation. The survey discussed key algorithms that ranged from classical methods to deep learning techniques, as well as the synergetic role of sensor fusion, convolutional neural networks (CNNs), SLAM, and high-definition maps in achieving robust autonomous navigation.

Yet, despite these gigantic advances, research in the area is still ongoing, addressing problems posed by occlusions, adverse weather conditions, and speed efficiency. The comparison between map-based and mapless navigation strategies sheds light on a tendency toward making the system more adaptive to real-time input while minimizing the dependency on prebuilt map specifications, augmented by improvements in SLAM techniques, including Kalman and particle filters, to increase localization accuracy in dynamic environments.

Future studies ought to develop farther efficient, smart, and big solutions combining real-time perception with virtually no computational expense. There should be a fruitful collaboration between academia and industry if we plan to deploy autonomous vehicles on a massive scale to address the limitations arising from existing systems and consequently operationalize complete, safe, and reliable systems of autonomous navigation.

## Figures and Tables

**Table 1:** Comparison of object detection architectures [18,22,23].

Architectures	# Parameters (M)	Frames per Second (FPS)	${\mathrm{AP}}^{\mathrm{COCO}}$
YOLOv5s	7.2	156	37.4
YOLOv7n	6.2	286	38.7
DETR	41	28	43.3
RT-DETR	32	114	53
Faster-RCNN	166	16	39

**Table 2:** The AP for each class and mAP evaluated based on the AP values of the classes.

Mitigating Technique	Faster R-CNN					YOLO-V3				
	V-AP	P-AP	TL-AP	TS-AP	mAP	V-AP	P-AP	TL-AP	TS-AP	mAP
None (clear conditions *)	72.61	40.99	26.07	38.12	44.45	76.57	37.12	46.22	50.56	52.62
None (rainy conditions **)	67.84	32.58	20.52	35.04	39.00	74.15	32.07	41.07	50.27	49.39
Deraining: DDN	67.00	28.55	20.02	35.55	37.78	73.07	29.89	40.05	48.74	47.94
Deraining: DeRaindrop	64.37	29.27	18.32	33.33	36.32	70.77	30.16	37.70	48.03	46.66
Deraining: PReNet	63.69	24.39	17.40	31.68	34.29	70.83	27.36	35.49	43.78	44.36
Image translation: UNIT	68.47	32.76	18.85	36.20	39.07	74.14	34.19	41.18	48.41	49.48
Domain adaptation	67.36	34.89	19.24	35.49	39.24	–	–	–	–	–

* Clear conditions represent non-rainy weather. ** Rainy conditions indicate adverse weather conditions.

**Table 3:** Breakdown of BDD dataset.

Time of Day	Label Count
Day	41,986
Night	31,900
Dusk/dawn	5942
Total	79,828

**Table 6:** Summary of types of maps used in autonomous systems with descriptions and key features.

Type of Map	Description	Key Features/Uses
Real-time (online) maps—SLAM	Build maps while tracking location in environments without preexisting maps or frequent changes.	Use sensors (cameras, LIDARs, range finders) for perception, mapping, and localization.
1. Topological maps (prebuilt maps) [52,53]	Focus on connectivity and relationships between locations rather than geometric details.	Represent environments as nodes and edges; useful for route planning and understanding layout.
2. Metric maps (prebuilt maps)	Provide detailed spatial information, including object distances and relationships.	Essential for precise navigation and obstacle avoidance.
2.1 Landmark-based maps (prebuilt maps) [54,55,56]	Use distinct environmental features (e.g., buildings, traffic signs) for localization and navigation.	Effective in GPS-denied environments.
2.2 Occupancy grid maps (prebuilt maps) [57]	Represent the environment as a grid of cells, each indicating the likelihood of being occupied.	Distinguishes free space from obstacles; crucial for perception and navigation.
2.2.1 Octree (prebuilt map) [58,59]	Hierarchical 3D grid system with eight-node subdivisions for spatial partitioning.	Efficient representation and processing of large 3D spaces.
2.2.2 CostMap (prebuilt map)	Assigns costs to cells based on traversal difficulty.	Guides safe and optimal paths; important for dynamic obstacle avoidance and path planning.
3. Geometric maps (prebuilt maps) [60,61]	Provide accurate geometrical data about objects and their locations.	Use vector data for high-precision tasks like obstacle avoidance and navigating through traffic.
High-accuracy maps—HD maps (prebuilt map) [62,63,64,65,66]	Offer fine resolution with centimeter-level precision, regularly updated for real-time accuracy.	Include detailed road environments, lane configurations, traffic signs, and obstacles; use sensors (LIDAR, GPS).

## Data Availability

Not applicable.

## References

[1] Redmon J., Farhadi A. YOLO9000: Better, faster, stronger. Proceedings of the IEEE Conference on Computer Vision and Pattern Recognition.

[2] Liu W., Anguelov D., Erhan D., Szegedy C., Reed S., Fu C.Y., Berg A.C., Leibe B., Matas J., Sebe N., Welling M. (2016). SSD: Single Shot MultiBox Detector. Proceedings of the Computer Vision—ECCV 2016.

[3] Ren S., He K., Girshick R., Sun J. (2016). Faster R-CNN: Towards real-time object detection with region proposal networks. IEEE Trans. Pattern Anal. Mach. Intell..

[4] Wojke N., Bewley A., Paulus D. Simple online and realtime tracking with a deep association metric. Proceedings of the 2017 IEEE International Conference on Image Processing (ICIP).

[5] Zhang Y., Sun P., Jiang Y., Yu D., Weng F., Yuan Z., Luo P., Liu W., Wang X. Bytetrack: Multi-object tracking by associating every detection box. Proceedings of the European Conference on Computer Vision.

[6] Vijayarajan V., Rajeshkannan R., Rajkumar Dhinakaran R. (2016). Automatidetection of moving objects using Kalman algorithm. Int. J. Pharm. Technol. IJPT.

[7] Aharon N., Orfaig R., Bobrovsky B.Z. (2022). BoT-SORT: Robust associations multi-pedestrian tracking. arXiv.

[8] Grisetti G., Kümmerle R., Stachniss C., Burgard W. (2010). A tutorial on graph-based SLAM. IEEE Intell. Transp. Syst. Mag..

[9] Liu F., Lu Z., Lin X. (2025). Vision-based environmental perception for autonomous driving. Proc. Inst. Mech. Eng. Part D J. Automob. Eng..

[10] Liang L., Ma H., Zhao L., Xie X., Hua C., Zhang M., Zhang Y. (2024). Vehicle Detection Algorithms for Autonomous Driving: A Review. Sensors.

[11] Turay T., Vladimirova T. (2022). Toward performing image classification and object detection with convolutional neural networks in autonomous driving systems: A survey. IEEE Access.

[12] Girshick R., Donahue J., Darrell T., Malik J. Rich feature hierarchies for accurate object detection and semantic segmentation. Proceedings of the IEEE Conference on Computer Vision and Pattern Recognition.

[13] He K., Zhang X., Ren S., Sun J. (2015). Spatial pyramid pooling in deep convolutional networks for visual recognition. IEEE Trans. Pattern Anal. Mach. Intell..

[14] Girshick R. (2015). Fast r-cnn. arXiv.

[15] Dai J., Li Y., He K., Sun J. (2016). R-fcn: Object detection via region-based fully convolutional networks. Adv. Neural Inf. Process. Syst..

[16] Lin T.Y., Dollár P., Girshick R., He K., Hariharan B., Belongie S. Feature pyramid networks for object detection. Proceedings of the IEEE Conference on Computer Vision and Pattern Recognition.

[17] Lin T.Y., Maire M., Belongie S., Hays J., Perona P., Ramanan D., Dollár P., Zitnick C.L. (2014). Microsoft coco: Common objects in context. Proceedings of the Computer Vision—ECCV 2014: 13th European Conference.

[18] Carion N., Massa F., Synnaeve G., Usunier N., Kirillov A., Zagoruyko S. (2020). End-to-end object detection with transformers. Proceedings of the European Conference on Computer Vision.

[19] Wu B., Iandola F., Jin P.H., Keutzer K. Squeezedet: Unified, small, low power fully convolutional neural networks for real-time object detection for autonomous driving. Proceedings of the IEEE Conference on Computer Vision and Pattern Recognition Workshops.

[20] Sandler M., Howard A., Zhu M., Zhmoginov A., Chen L.C. Mobilenetv2: Inverted residuals and linear bottlenecks. Proceedings of the IEEE Conference on Computer Vision and Pattern Recognition.

[21] Duan K., Bai S., Xie L., Qi H., Huang Q., Tian Q. CenterNet: Keypoint Triplets for Object Detection. Proceedings of the IEEE/CVF International Conference on Computer Vision (ICCV).

[22] Zhao Y., Lv W., Xu S., Wei J., Wang G., Dang Q., Liu Y., Chen J. Detrs beat yolos on real-time object detection. Proceedings of the IEEE/CVF Conference on Computer Vision and Pattern Recognition.

[23] Wang C.Y., Bochkovskiy A., Liao H.Y.M. YOLOv7: Trainable bag-of-freebies sets new state-of-the-art for real-time object detectors. Proceedings of the IEEE/CVF Conference on Computer Vision and Pattern Recognition.

[24] Hnewa M., Radha H. (2020). Object detection under rainy conditions for autonomous vehicles: A review of state-of-the-art and emerging techniques. IEEE Signal Process. Mag..

[25] Qian R., Tan R.T., Yang W., Su J., Liu J. Attentive generative adversarial network for raindrop removal from a single image. Proceedings of the IEEE Conference on Computer Vision and Pattern Recognition.

[26] Ren D., Zuo W., Hu Q., Zhu P., Meng D. Progressive image deraining networks: A better and simpler baseline. Proceedings of the IEEE/CVF Conference on Computer Vision and Pattern Recognition.

[27] Chen Y., Li W., Sakaridis C., Dai D., Van Gool L. Domain adaptive faster r-cnn for object detection in the wild. Proceedings of the IEEE Conference on Computer Vision and Pattern Recognition.

[28] Lin H., Parsi A., Mullins D., Horgan J., Ward E., Eising C., Denny P., Deegan B., Glavin M., Jones E. (2024). A Study on Data Selection for Object Detection in Various Lighting Conditions for Autonomous Vehicles. J. Imaging.

[29] Yu F., Chen H., Wang X., Xian W., Chen Y., Liu F., Madhavan V., Darrell T. Bdd100k: A diverse driving dataset for heterogeneous multitask learning. Proceedings of the IEEE/CVF Conference on Computer Vision and Pattern Recognition.

[30] Brostow G.J., Fauqueur J., Cipolla R. (2009). Semantic object classes in video: A high-definition ground truth database. Pattern Recognit. Lett..

[31] Geiger A., Lenz P., Stiller C., Urtasun R. (2013). Vision meets robotics: The kitti dataset. Int. J. Robot. Res..

[32] Cordts M., Omran M., Ramos S., Rehfeld T., Enzweiler M., Benenson R., Franke U., Roth S., Schiele B. The cityscapes dataset for semantic urban scene understanding. Proceedings of the IEEE Conference on Computer Vision and Pattern Recognition.

[33] Varma G., Subramanian A., Namboodiri A., Chandraker M., Jawahar C. IDD: A dataset for exploring problems of autonomous navigation in unconstrained environments. Proceedings of the 2019 IEEE Winter Conference on Applications of Computer Vision (WACV).

[34] Neuhold G., Ollmann T., Rota Bulo S., Kontschieder P. The mapillary vistas dataset for semantic understanding of street scenes. Proceedings of the IEEE International Conference on Computer Vision.

[35] Ros G., Sellart L., Materzynska J., Vazquez D., Lopez A.M. The synthia dataset: A large collection of synthetic images for semantic segmentation of urban scenes. Proceedings of the IEEE Conference on Computer Vision and Pattern Recognition.

[36] Richter S.R., Hayder Z., Koltun V. Playing for benchmarks. Proceedings of the IEEE International Conference on Computer Vision.

[37] Huang X., Cheng X., Geng Q., Cao B., Zhou D., Wang P., Lin Y., Yang R. The apolloscape dataset for autonomous driving. Proceedings of the IEEE Conference on Computer Vision and Pattern Recognition Workshops.

[38] Sun P., Kretzschmar H., Dotiwalla X., Chouard A., Patnaik V., Tsui P., Guo J., Zhou Y., Chai Y., Caine B. Scalability in perception for autonomous driving: Waymo open dataset. Proceedings of the IEEE/CVF Conference on Computer Vision and Pattern Recognition.

[39] Tithi J.J., Aananthakrishnan S., Petrini F. (2020). Online and Real-time Object Tracking Algorithm with Extremely Small Matrices. arXiv.

[40] Bewley A., Ge Z., Ott L., Ramos F., Upcroft B. Simple online and realtime tracking. Proceedings of the 2016 IEEE International Conference on Image Processing (ICIP).

[41] Sridhar V.H., Roche D.G., Gingins S. (2019). Tracktor: Image-based automated tracking of animal movement and behaviour. Methods Ecol. Evol..

[42] Abouelyazid M. (2023). Comparative Evaluation of SORT, DeepSORT, and ByteTrack for Multiple Object Tracking in Highway Videos. Int. J. Sustain. Infrastruct. Cities Soc..

[43] Li Y., Xiao Z., Yang L., Meng D., Zhou X., Fan H., Zhang L. (2024). AttMOT: Improving multiple-object tracking by introducing auxiliary pedestrian attributes. IEEE Trans. Neural Netw. Learn. Syst..

[44] Alarcon N. (2022). DRIVE Labs: How Localization Helps Vehicles Find Their Way | NVIDIA Technical Blog. https://developer.nvidia.com/blog/drive-labs-how-localization-helps-vehicles-find-their-way/.

[45] Azzam R., Taha T., Huang S., Zweiri Y. (2020). Feature-based visual simultaneous localization and mapping: A survey. SN Appl. Sci..

[46] Agostinho L.R., Ricardo N.M., Pereira M.I., Hiolle A., Pinto A.M. (2022). A practical survey on visual odometry for autonomous driving in challenging scenarios and conditions. IEEE Access.

[47] Abdelaziz N., El-Rabbany A. (2023). INS/LIDAR/Stereo SLAM Integration for Precision Navigation in GNSS-Denied Environments. Sensors.

[48] Yin H., Xu X., Lu S., Chen X., Xiong R., Shen S., Stachniss C., Wang Y. (2024). A survey on global lidar localization: Challenges, advances and open problems. Int. J. Comput. Vis..

[49] Wang H., Yin Y., Jing Q. (2023). Comparative analysis of 3D LiDAR scan-matching methods for state estimation of autonomous surface vessel. J. Mar. Sci. Eng..

[50] Golledge R.G., Gärling T. (2004). Cognitive maps and urban travel. Handbook of Transport Geography and Spatial Systems.

[51] Epstein R.A., Patai E.Z., Julian J.B., Spiers H.J. (2017). The cognitive map in humans: Spatial navigation and beyond. Nat. Neurosci..

[52] Qi Y., Wang R., He B., Lu F., Xu Y. (2022). Compact and efficient topological mapping for large-scale environment with pruned Voronoi diagram. Drones.

[53] Rawlinson D., Jarvis R. (2008). Topologically-directed navigation. Robotica.

[54] Murali V., Chiu H.P., Samarasekera S., Kumar R.T. Utilizing semantic visual landmarks for precise vehicle navigation. Proceedings of the 2017 IEEE 20th International Conference on Intelligent Transportation Systems (ITSC).

[55] Levinson J., Montemerlo M., Thrun S. Map-based precision vehicle localization in urban environments. Proceedings of the Robotics: Science and Systems.

[56] Sundar K., Srinivasan S., Misra S., Rathinam S., Sharma R. Landmark Placement for Localization in a GPS-denied Environment. Proceedings of the 2018 Annual American Control Conference (ACC).

[57] Li Y., Ruichek Y. (2014). Occupancy grid mapping in urban environments from a moving on-board stereo-vision system. Sensors.

[58] Hornung A., Wurm K.M., Bennewitz M., Stachniss C., Burgard W. (2013). OctoMap: An efficient probabilistic 3D mapping framework based on octrees. Auton. Robot..

[59] Leven J., Corso J., Cohen J., Kumar S. Interactive visualization of unstructured grids using hierarchical 3D textures. Proceedings of the Symposium on Volume Visualization and Graphics.

[60] Lafarge F., Mallet C. (2012). Creating large-scale city models from 3D-point clouds: A robust approach with hybrid representation. Int. J. Comput. Vis..

[61] Wolf D., Howard A., Sukhatme G.S. Towards geometric 3D mapping of outdoor environments using mobile robots. Proceedings of the 2005 IEEE/RSJ International Conference on Intelligent Robots and Systems.

[62] Ebrahimi Soorchaei B., Razzaghpour M., Valiente R., Raftari A., Fallah Y.P. (2022). High-definition map representation techniques for automated vehicles. Electronics.

[63] Elghazaly G., Frank R., Harvey S., Safko S. (2023). High-definition maps: Comprehensive survey, challenges and future perspectives. IEEE Open J. Intell. Transp. Syst..

[64] Asrat K.T., Cho H.J. (2024). A Comprehensive Survey on High-Definition Map Generation and Maintenance. ISPRS Int. J. Geo-Inf..

[65] Charroud A., El Moutaouakil K., Palade V., Yahyaouy A., Onyekpe U., Eyo E.U. (2024). Localization and Mapping for Self-Driving Vehicles: A Survey. Machines.

[66] Wong K., Gu Y., Kamijo S. (2020). Mapping for autonomous driving: Opportunities and challenges. IEEE Intell. Transp. Syst. Mag..

[67] Li T., Zhang H., Gao Z., Chen Q., Niu X. (2018). High-accuracy positioning in urban environments using single-frequency multi-GNSS RTK/MEMS-IMU integration. Remote Sens..

[68] Ma H., Zhao Q., Verhagen S., Psychas D., Liu X. (2020). Assessing the performance of multi-GNSS PPP-RTK in the local area. Remote Sens..

[69] Aldibaja M., Suganuma N., Yoneda K., Yanase R. (2022). Challenging environments for precise mapping using GNSS/INS-RTK systems: Reasons and analysis. Remote Sens..

[70] Gargoum S.A., Basyouny K.E. (2019). A literature synthesis of LiDAR applications in transportation: Feature extraction and geometric assessments of highways. GISci. Remote Sens..

[71] Blochliger F., Fehr M., Dymczyk M., Schneider T., Siegwart R. Topomap: Topological mapping and navigation based on visual slam maps. Proceedings of the 2018 IEEE International Conference on Robotics and Automation (ICRA).

[72] Drouilly R., Rives P., Morisset B. Semantic representation for navigation in large-scale environments. Proceedings of the 2015 IEEE International Conference on Robotics and Automation (ICRA).

[73] Kumpakeaw S., Dillmann R. (2007). Semantic road maps for autonomous vehicles. Proceedings of the Autonome Mobile Systeme 2007: 20. Fachgespräch Kaiserslautern.

[74] Map Rendering | Mapping Technology | Platform | HERE. https://www.here.com/platform/map-rendering.

[75] HD Map | TomTom. https://www.tomtom.com/products/orbis-maps-for-automation/.

[76] Berrio J.S., Ward J., Worrall S., Nebot E. Identifying robust landmarks in feature-based maps. Proceedings of the 2019 IEEE Intelligent Vehicles Symposium (IV).

[77] Kim C., Cho S., Sunwoo M., Jo K. (2018). Crowd-sourced mapping of new feature layer for high-definition map. Sensors.

[78] Scholtes M., Westhofen L., Turner L.R., Lotto K., Schuldes M., Weber H., Wagener N., Neurohr C., Bollmann M.H., Körtke F. (2021). 6-Layer Model for a Structured Description and Categorization of Urban Traffic and Environment. IEEE Access.

[79] Stepanyants V., Romanov A. An Object-Oriented Approach to a Structured Description of Machine Perception and Traffic Participant Interactions in Traffic Scenarios. Proceedings of the 2022 IEEE 7th International Conference on Intelligent Transportation Engineering (ICITE).

[80] Elhashash M., Albanwan H., Qin R. (2022). A review of mobile mapping systems: From sensors to applications. Sensors.

[81] Chang Y.F., Chiang K.W., Tsai M.L., Lee P.L., Zeng J.C., El-Sheimy N., Darweesh H. (2023). The implementation of semi-automated road surface markings extraction schemes utilizing mobile laser scanned point clouds for HD maps production. Int. Arch. Photogramm. Remote Sens. Spat. Inf. Sci..

[82] Peng C.W., Hsu C.C., Wang W.Y. (2020). Cost effective mobile mapping system for color point cloud reconstruction. Sensors.

[83] Ilci V., Toth C. (2020). High definition 3D map creation using GNSS/IMU/LiDAR sensor integration to support autonomous vehicle navigation. Sensors.

[84] Ben Elallid B., Benamar N., Senhaji Hafid A., Rachidi T., Mrani N. (2022). A Comprehensive Survey on the Application of Deep and Reinforcement Learning Approaches in Autonomous Driving. J. King Saud Univ.-Comput. Inf. Sci..

[85] Girardeau-Montaut D. CloudCompare—Open Source Project—danielgm.net. https://www.danielgm.net/cc/.

[86] Gholami Shahbandi S., Magnusson M. (2019). 2D map alignment with region decomposition. Auton. Robot..

[87] Xu S., Wang R., Zheng H. (2016). Road curb extraction from mobile LiDAR point clouds. IEEE Trans. Geosci. Remote Sens..

[88] Kumar P., McElhinney C.P., Lewis P., McCarthy T. (2013). An automated algorithm for extracting road edges from terrestrial mobile LiDAR data. ISPRS J. Photogramm. Remote Sens..

[89] Kuang H., Wang B., An J., Zhang M., Zhang Z. (2020). Voxel-FPN: Multi-scale voxel feature aggregation for 3D object detection from LIDAR point clouds. Sensors.

[90] Li Y., Olson E.B. Extracting general-purpose features from LIDAR data. Proceedings of the 2010 IEEE International Conference on Robotics and Automation.

[91] Yin R., Cheng Y., Wu H., Song Y., Yu B., Niu R. (2020). Fusionlane: Multi-sensor fusion for lane marking semantic segmentation using deep neural networks. IEEE Trans. Intell. Transp. Syst..

[92] Tian W., Yu X., Hu H. (2023). Interactive attention learning on detection of lane and lane marking on the road by monocular camera image. Sensors.

[93] Zhao X., Sun P., Xu Z., Min H., Yu H. (2020). Fusion of 3D LIDAR and camera data for object detection in autonomous vehicle applications. IEEE Sens. J..

[94] Zhao L., Zhou H., Zhu X., Song X., Li H., Tao W. (2023). Lif-seg: Lidar and camera image fusion for 3d lidar semantic segmentation. IEEE Trans. Multimed..

[95] Lagahit M.L.R., Matsuoka M. (2023). Focal Combo Loss for Improved Road Marking Extraction of Sparse Mobile LiDAR Scanning Point Cloud-Derived Images Using Convolutional Neural Networks. Remote Sens..

[96] Huang A.S., Moore D., Antone M., Olson E., Teller S. (2009). Finding multiple lanes in urban road networks with vision and lidar. Auton. Robot..

[97] Zheng C., Cao X., Tang K., Cao Z., Sizikova E., Zhou T., Li E., Liu A., Zou S., Yan X. (2023). High-definition map automatic annotation system based on active learning. AI Mag..

[98] Li Q., Wang Y., Wang Y., Zhao H. Hdmapnet: An online hd map construction and evaluation framework. Proceedings of the 2022 International Conference on Robotics and Automation (ICRA).

[99] Elhousni M., Lyu Y., Zhang Z., Huang X. Automatic building and labeling of hd maps with deep learning. Proceedings of the AAAI Conference on Artificial Intelligence.

[100] He L., Jiang S., Liang X., Wang N., Song S. Diff-net: Image feature difference based high-definition map change detection for autonomous driving. Proceedings of the 2022 International Conference on Robotics and Automation (ICRA).

[101] Zhang P., Zhang M., Liu J. (2021). Real-time HD map change detection for crowdsourcing update based on mid-to-high-end sensors. Sensors.

[102] Jo K., Kim C., Sunwoo M. (2018). Simultaneous localization and map change update for the high definition map-based autonomous driving car. Sensors.

[103] Alonso I.P., Llorca D.F.F., Gavilan M., Pardo S.Á.Á., García-Garrido M.Á., Vlacic L., Sotelo M.Á. (2012). Accurate global localization using visual odometry and digital maps on urban environments. IEEE Trans. Intell. Transp. Syst..

[104] Kang J.M., Yoon T.S., Kim E., Park J.B. (2020). Lane-level map-matching method for vehicle localization using GPS and camera on a high-definition map. Sensors.

[105] Vargas J., Alsweiss S., Toker O., Razdan R., Santos J. (2021). An overview of autonomous vehicles sensors and their vulnerability to weather conditions. Sensors.

[106] Wang W., You X., Chen L., Tian J., Tang F., Zhang L. (2022). A scalable and accurate de-snowing algorithm for LiDAR point clouds in winter. Remote Sens..

[107] Tsushima F., Kishimoto N., Okada Y., Che W. (2020). Creation of high definition map for autonomous driving. Int. Arch. Photogramm. Remote Sens. Spat. Inf. Sci..

[108] Zhang H., Venkatramani S., Paz D., Li Q., Xiang H., Christensen H.I. (2023). Probabilistic semantic mapping for autonomous driving in urban environments. Sensors.

[109] Zhang F., Shi W., Chen M., Huang W., Liu X. (2023). Open HD map service model: An interoperable high-Definition map data model for autonomous driving. Int. J. Digit. Earth.

[110] Ma W.C., Tartavull I., Bârsan I.A., Wang S., Bai M., Mattyus G., Homayounfar N., Lakshmikanth S.K., Pokrovsky A., Urtasun R. Exploiting sparse semantic HD maps for self-driving vehicle localization. Proceedings of the 2019 IEEE/RSJ International Conference on Intelligent Robots and Systems (IROS).

[111] Barsi A., Poto V., Somogyi A., Lovas T., Tihanyi V., Szalay Z. (2017). Supporting autonomous vehicles by creating HD maps. Prod. Eng. Arch..

[112] Taeihagh A., Lim H.S.M. (2019). Governing autonomous vehicles: Emerging responses for safety, liability, privacy, cybersecurity, and industry risks. Transp. Rev..

[113] Linkov V., Zámečník P., Havlíčková D., Pai C.W. (2019). Human factors in the cybersecurity of autonomous vehicles: Trends in current research. Front. Psychol..

[114] Parkinson S., Ward P., Wilson K., Miller J. (2017). Cyber threats facing autonomous and connected vehicles: Future challenges. IEEE Trans. Intell. Transp. Syst..

[115] Chattopadhyay A., Lam K.Y., Tavva Y. (2020). Autonomous vehicle: Security by design. IEEE Trans. Intell. Transp. Syst..

[116] Lee S., Ryu J.H. (2024). Autonomous Vehicle Localization Without Prior High-Definition Map. IEEE Trans. Robot..

[117] Shaviv I. (2024). Benefits of Mapless Autonomous Driving Technology.

[118] Guzel M.S., Bicker R. (2012). A behaviour-based architecture for mapless navigation using vision. Int. J. Adv. Robot. Syst..

[119] Xue H., Hein B., Bakr M., Schildbach G., Abel B., Rueckert E. (2022). Using deep reinforcement learning with automatic curriculum learning for mapless navigation in intralogistics. Appl. Sci..

[120] Wang N., Wang Y., Zhao Y., Wang Y., Li Z. (2022). Sim-to-real: Mapless navigation for USVs using deep reinforcement learning. J. Mar. Sci. Eng..

[121] Pavel M.I., Tan S.Y., Abdullah A. (2022). Vision-based autonomous vehicle systems based on deep learning: A systematic literature review. Appl. Sci..

[122] Baten S., Lutzeler M., Dickmanns E.D., Mandelbaum R., Burt P.J. (1998). Techniques tor autonomous, off-road navigation. IEEE Intell. Syst. Their Appl..

[123] Taketomi T., Uchiyama H., Ikeda S. (2017). Visual SLAM algorithms: A survey from 2010 to 2016. IPSJ Trans. Comput. Vis. Appl..

[124] Civera J., Davison A.J., Montiel J.M. (2008). Inverse depth parametrization for monocular SLAM. IEEE Trans. Robot..

[125] Eade E., Drummond T. Scalable monocular SLAM. Proceedings of the 2006 IEEE Computer Society Conference on Computer Vision and Pattern Recognition (CVPR’06).

[126] Klein G., Murray D. Parallel tracking and mapping for small AR workspaces. Proceedings of the 2007 6th IEEE and ACM International Symposium on Mixed and Augmented Reality.

[127] Mur-Artal R., Montiel J.M.M., Tardos J.D. (2015). ORB-SLAM: A versatile and accurate monocular SLAM system. IEEE Trans. Robot..

[128] Schlegel D., Colosi M., Grisetti G. Proslam: Graph slam from a programmer’s perspective. Proceedings of the 2018 IEEE International Conference on Robotics and Automation (ICRA).

[129] Sumikura S., Shibuya M., Sakurada K. OpenVSLAM: A versatile visual SLAM framework. Proceedings of the 27th ACM International Conference on Multimedia.

[130] Campos C., Elvira R., Rodríguez J.J.G., Montiel J.M., Tardós J.D. (2021). Orb-slam3: An accurate open-source library for visual, visual–inertial, and multimap slam. IEEE Trans. Robot..

[131] Giubilato R., Pertile M., Debei S. A comparison of monocular and stereo visual FastSLAM implementations. Proceedings of the 2016 IEEE Metrology for Aerospace (MetroAeroSpace).

[132] Ullah I., Su X., Zhang X., Choi D. (2020). Simultaneous localization and mapping based on Kalman filter and extended Kalman filter. Wirel. Commun. Mob. Comput..

[133] Saman A.B.S.H., Lotfy A.H. An implementation of SLAM with extended Kalman filter. Proceedings of the 2016 6th International Conference on Intelligent and Advanced Systems (ICIAS).

[134] Cadena C., Carlone L., Carrillo H., Latif Y., Scaramuzza D., Neira J., Reid I., Leonard J.J. (2016). Past, present, and future of simultaneous localization and mapping: Toward the robust-perception age. Robot. Auton. Syst..

[135] Liu T., Xu C., Qiao Y., Jiang C., Yu J. (2024). Particle Filter SLAM for Vehicle Localization. arXiv.

[136] Montemerlo M., Thrun S., Koller D., Wegbreit B. FastSLAM: A factored solution to the simultaneous localization and mapping problem. Proceedings of the AAAI Conference on Artificial Intelligence.

[137] Montemerlo M., Thrun S., Koller D., Wegbreit B. (2003). FastSLAM 2.0: An improved particle filtering algorithm for simultaneous localization and mapping that provably converges. Proc. Int. Jt. Conf. Artif. Intell..

[138] Song W., Yang Y., Fu M., Kornhauser A., Wang M. (2018). Critical Rays Self-adaptive Particle Filtering SLAM. J. Intell. Robot. Syst..

